# Impaired B Cell Apoptosis Results in Autoimmunity That Is Alleviated by Ablation of Btk

**DOI:** 10.3389/fimmu.2021.705307

**Published:** 2021-08-26

**Authors:** Jacqueline A. Wright, Cassandra Bazile, Emily S. Clark, Gianluca Carlesso, Justin Boucher, Eden Kleiman, Tamer Mahmoud, Lily I. Cheng, Darlah M. López-Rodríguez, Anne B. Satterthwaite, Norman H. Altman, Eric L. Greidinger, Wasif N. Khan

**Affiliations:** ^1^Department of Microbiology and Immunology, Miller School of Medicine, University of Miami, Miami, FL, United States; ^2^Early Oncology Discovery, Early Oncology R&D, AstraZeneca, Gaithersburg, MD, United States; ^3^Oncology Safety/Pathology, Clinical Pharmacology and Safety Sciences, AstraZeneca, Gaithersburg, MD, United States; ^4^Department of Immunology, University of Texas Southwestern Medical Center, Dallas, TX, United States; ^5^Department of Pathology, Miller School of Medicine, University of Miami, Miami, FL, United States; ^6^Department of Medicine, Miller School of Medicine, University of Miami, Miami, FL, United States

**Keywords:** autoimmunity, apoptosis, Bcl-2-like 11 (Bim), B cell tolerance, systemic lupus - erythematosus, autoantibodies, Sjogren’s syndrome, Bruton’s tyrosine kinase (Bkt)

## Abstract

While apoptosis plays a role in B-cell self-tolerance, its significance in preventing autoimmunity remains unclear. Here, we report that dysregulated B cell apoptosis leads to delayed onset autoimmune phenotype in mice. Our longitudinal studies revealed that mice with B cell-specific deletion of pro-apoptotic Bim (*BBim^fl/fl^*) have an expanded B cell compartment with a notable increase in transitional, antibody secreting and recently described double negative (DN) B cells. They develop greater hypergammaglobulinemia than mice lacking Bim in all cells and accumulate several autoantibodies characteristic of Systemic Lupus Erythematosus (SLE) and related Sjögren’s Syndrome (SS) including anti-nuclear, anti-Ro/SSA and anti-La/SSB at a level comparable to NODH2h4 autoimmune mouse model. Furthermore, lymphocytes infiltrated the tissues including submandibular glands and formed follicle-like structures populated with B cells, plasma cells and T follicular helper cells indicative of ongoing immune reaction. This autoimmunity was ameliorated upon deletion of Bruton’s tyrosine kinase (Btk) gene, which encodes a key B cell signaling protein. These studies suggest that Bim-mediated apoptosis suppresses and B cell tyrosine kinase signaling promotes B cell-mediated autoimmunity.

## Introduction

A vast B cell repertoire is generated by a random recombination of exons that are assembled to encode a diverse set of B cell receptors (BCRs) necessary to mount antigen-specific immune responses against a large variety of pathogen-derived antigens as well as potential neoantigens. The process called V(D)J recombination inherently produces a large number of self-reactive B cell clones. Therefore, exquisitely controlled mechanisms of immune tolerance operate during development in the bone marrow (BM) to eliminate autoreactive clones (1, 2). The primary mechanisms of tolerance in the BM include receptor editing, clonal deletion by apoptosis and anergy. Central tolerance eliminates most of the autoreactive B cells immediately after completing and displaying their assembled BCRs. Additional tolerance checkpoints purge the autoreactive B cell clones that variably escape these controls in the BM or are generated in the germinal centers during an immune response (3, 4).

While relatively strong BCR signaling results in the immature B cell negative selection, tonic or low level BCR signaling is continuously required for the survival and maturation of newly formed immature B cells in the BM as well as after their emigration to the spleen as transitional 1 (T1) B cells (5–9). We and others have shown that B cells at the T1 stage remain sensitive to apoptosis to serve as a second tolerance check point allowing deletion of autoreactive B cells in the periphery (7–9). These studies also showed that the window of opportunity for self-tolerance is limited as progression into transitional 2 (T2) B cells allows BCR engagement to promote positive selection (7, 10–13). BCR signaling in T2 cells induces sustained NF-kB activation, upregulation of BAFFR (TNFRSF13c) and more robust synergy between BCR and BAFFR. Excess availability of BAFF and increased BAFFR signaling can sway BCR-engaged autoreactive transitional B cell clones to undergo maturation into follicular (Fo) and marginal zone (MZ) B cells and can promote autoimmunity (6, 7, 14–17). BAFF function becomes particularly important when other B cell coreceptors positively influence autoreactive B cell activation (5, 6, 14, 16, 18). For example, self RNA and DNA reactive B cell clones receive the first antigen specific signal *via* the BCRs, which endocytose nucleic acids and deliver them to endosomal TLR7 or TLR9. TLRs can then provide the second signal to activate autoreactive B cells (19–23). Availability of BAFF can enhance positive selection of BCR and TLR activated autoreactive B cells and promote their maturation. Thus, TLR and BAFFR can synergize to dysregulate autoreactive BCR signaling towards B cell survival and maturation (18). Recent studies with systemic lupus erythematosus (SLE) patients have identified polymorphisms in genes that dysregulate signaling downstream of BCR, BAFFR and TLRs, supporting synergy between these receptors can promote positive selection of autoreactive B cells leading to autoimmunity (18, 24–26).

Importantly, autoimmune diseases associated with autoAbs are highly common in humans. In fact, both SLE and SS involve B cell hyperactivity that contributes to the development of autoimmune disease manifestation (27). However, whether or not apoptosis-mediated B cell immune tolerance prevents autoimmune disease is an active area of research (16, 28, 29). Clonal deletion of autoreactive B cells by apoptosis can be mediated by cell-extrinsic Fas/FasL – dependent pathway and cell intrinsic mechanisms controlled by pro-apoptotic members of the Bcl-2 family (30, 31). In fact, both pathways may eventually converge on the executioner caspases to induce cell death. Therefore, dysregulated apoptosis is a contributing factor to the escape of autoreactive B cells. However, the physiological role for cell intrinsic pathways, including BH3-only proapoptotic members of the Bcl-2 family, such as Bcl-2-like 11 (Bim) remains elusive (32, 33). Bim is particularly critical in facilitating apoptosis when autoreactive B cells are destined to die (34). Although genetic alterations have been made in apoptosis pathways that variably promote SLE-like disease including *Bim^-/-^* (C57BL/6 X 129Ssv) (35), B6.Fas^lpr/lpr^ and B6.Bim^-/-^.Fas^lpr/lpr^ (31, 36), the contributions of specific immune cells lacking Bim to autoimmune pathology are not yet fully elucidated. Global deletion of Bim (*Bim^-/-^)* results in SLE-like autoimmune disease in mice with splenomegaly and lymphadenopathy accompanied with lymphocyte infiltration and autoantibody production on a mixed C57BL/6x129Sv genetic background (35). Because Bim is also required for negative selection of T cells at multiple checkpoints and in myeloid cells, the contribution of apoptosis regulation in specific cell types to autoimmune pathology in Bim^-/-^ mice remained unknown. Subsequent adaptive transfer experiments demonstrated that Bim-deficient dendritic cells (DCs) can drive autoimmune pathology (37, 38). More recently, conditional gene deletion approach was used to demonstrate that myeloid cell-specific deletion of Bim led to a severe SLE disease whereas they noted CD4 T cell- or B cell-specific Bim gene deletion did not result in significant manifestation of autoimmunity (39). Another excellent study interrogating Bim function in B cells, did not observe autoimmune phenotype and focused on B cell lymphoma genesis (39, 40). However detailed B cell analysis, particularly relating to autoimmunity was not shown in either study (39, 40). Cumulatively, prior studies implicate Bim in suppressing autoimmunity mediated by myeloid and dendritic cells whereas its role in restraining T and B cells in promoting autoimmune disease remains work in progress.

Here we report detailed longitudinal studies in mice with CD19-Cre mediated B cell-specific Bim deletion the revealing a delayed onset SLE/SS-like autoimmune disease in C57BL/6 background. The *BBim^fl/fl^* mice had more exaggerated B cell expansion than the Bim null mice, which display a mild autoimmunity in the C57BL/6 background, whereas the increase in T cell numbers was comparable. Autoantibody production included Anti-dsDNA, Anti-SSA, anti-SSB autoantibodies (autoAbs) characteristic of SLE and SS, and their levels were comparable or exceeded autoantibody levels in age-matched NODH2h4 mice. These autoimmune phenotypes were accompanied with lymphocyte infiltration of salivary submandibular glands (SG) which were populated by B cell, Tfh and plasma cells. Thus, Bim-mediated B cell apoptosis suppresses a wide range of autoAbs production and dysregulated apoptosis in B cells promotes T cell activation and participation in autoimmunity. The autoimmunity in *BBim^fl/fl^* mice was remediated by deletion of Btk, a key B cell signaling tyrosine kinase, suggesting contribution of altered B cell signaling to autoimmune pathology supporting utility of Btk and tyrosine kinase inhibitors in autoimmunity (41, 42).

## Methods

### Mice

Adult B57BL/6 (B6) mice were originally obtained from Jackson Laboratory, Bar Harbor, ME and were subsequently bred in house. Bim^fl/fl^ mice were generated by Korsmeyer group (43) and were obtained from S. Zinkel, Vanderbilt University. B lineage specific Bim-deficient mice were generated by intercrossing Bim^fl/fl^ mice with CD19^cre^ mice (44). All B*Bim^fl/fl^* used were heterozygous for CD19^cre^. It has been shown that mice expressing CD19^cre^ have no discernable effect on B cell development (44). The original *Bim-/-* mice in *B6.129S1-Bcl2l11tm1.1Ast/J* ^56^ had been backcrossed into C57BL/6J (Jackson Laboratory). Spleens from the Bim-deficient mice were kindly provided by Richard T Libby, Flaum Eye Institute, University of Rochester Medical Center, referred here as *Bim-/-*. The generation of Btk^-/-^ mice have been previously described (45). Mice were treated humanely in accordance with federal, state, and institutional guidelines.

### Cell Isolation and *In Vitro* Culture Conditions

Spleens were removed and mechanically disrupted, generating a single cell suspension. Red blood cells were then lysed using RBC lysis buffer (Biolegend) according to manufacturer’s instructions. Splenic B cells were enriched by negative selection to avoid inadvertent activation, either by autoMACS depletion using anti-CD43 microbeads (Miltenyi Biotec) or using anti-CD43 microbeads B cell enrichment kit (BD Biosciences). B cell purity was determined to be between 92-98% as determined by flow cytometric analysis using antibodies directed against CD19 and IgM (**Table 1**). For B cell proliferation assays, purified B cells were cultured in RPMI (Hyclone laboratories) supplemented with 10% fetal calf serum, 55nM β-mercaptoethanol, 2nM L-glutamine and 100IU penicillin/streptomycin in a 37°C humidified incubator. *In vitro* cultures were left nonstimulated or treated with F(ab’)_2_ goat anti-mouse IgM (10μg/ml; Jackson ImmunoResearch Laboratories), recombinant human BAFF purified from Chinese Hamster ovary cells (46) (100ng/ml), anti-CD40 (2.5μg/ml; BD Bioscience), LPS (2.5μg/ml Sigma- Aldrich), or CpG (1.0μg/ml) at the times indicated. To measure cell death, purified B cells were stimulated with LPS (1μg/ml), CPG (ODN-1826 1μg/ml; InvivoGen), CL097 (1μg/ml; InvivoGen), or Fa(b’)_2_ goat anti-mouse IgM (1μg/ml) in the absence or in the presence of IL-21 (25ng/ml; RnD System) for 48 hours at 37°C. To assess cytokine production, total splenocytes were stimulated with CPG or CL097 for 24 hours and PMA (50ng/ml) and ionomycin (500ng/ml) were added to the culture for the last 4 hours.

**Table 1:** Antibodies used in this study.

Ab	Conjugate	Clone	Manufacturer	Catalog No.	Dilution
**B cell antibodies**					
**AA4**	PE-Cy7	AA4.1	Biolegend	136506	100x
**B220**	V500	RA3-6B2	BD Bioscience	561226	200x
**CD19**	PE-CF594	ID3	BD Bioscience	562291	200x
**CD21**	PE	7g6	BD Bioscience	552957	200x
**CD23**	PerCP-CY5.5	3B4	Biolegend	101618	200x
**CD138**	APC	281-2	Biolegend	142506	100x
**IgD**	Pac Blue	11-26c.2a	Biolegend	405711	400x
**IgM**	BV711	ll/41	BD Bioscience	743327	400x
**T cell antibodies**					
**CD3**	BV605	ucht1	Biolegend	300459	100x
**CD4**	BV711	GK1.5	BD Bioscience	563050	100x
**CD8**	BV605	53-6.7	BD Bioscience	563152	100x
**CD44**	PE	im7	eBioscience	12-0441-83	200x
CD62L	Alexa Fluor 700	mel-14	eBioscience	56-0621-82	200x
**Cytokine, proliferation and viability antibodies**					
**Annexin V**	FITC	–	Biolegend	640922	–
**7AAD**	–	–	Biolegend	640922	–
**IFNα**	FITC	RMMA-1	PBL assay science	22100-3	100x
**IFNβ**	FITC	MMHB-3	PBL assay science	21400-3	100x
**IFNγ**	BV786	Q31-378	BD Bioscience	564723	100x
**IL-6**	PE	MP5-20F3	MP5-20F3	504504	100x
**L-10**	BV605	JES5-16E3	BD Bioscience	564082	100x
**Ki-67**	PE-Cy7	B56	BD Bioscience	561283	100x
**Immunohistochemistry Antibodies**					
Goat anti-mouse CD3	–	M20	Santa Cruz Biotechnology	SC-1127	100x
CD19	Biotin	ID3	BD Bioscience	553784	100x
Anti-goat	Alexa Fluor 647	–	Life Technologies	A-21447	1000x
Streptavidin	Alexa Fluor 488	–	Biolegend	405235	100x

### Flow Cytometry

For phenotypic analysis, single-cell suspensions were prepared from spleens, inguinal lymph nodes, submandibular lymph nodes, tertiary lymphoid structures (TLS) and the blood of WT, B*Bim^fl/fl^*, Bim^−/−^, Btk^−/−^ and B*Bim^fl/fl^* × Btk^−/−^ mice. Cells were stained with fluorescently labelled antibodies in various combinations to identify; B cells and subpopulations including T1, MZ, pMZ, An1, CD21lo and, CD4 and CD8 T cells and their subpopulations including T follicular cells, as defined below. Antibodies, fluorochrome labelling and sources are detailed in **Table 1** and indicated in figure legends.

Intracellular cytokine staining was carried out by first staining cell surface markers in PBS with 2% serum after incubation with FcR-block (CD16), washed and stained with antibodies to various cytokines (IFN-γ, IFN-α, IL-6, IL-10 and TNF-α) using the BD Biosciences fix/perm kit. Dead cells and doublets were excluded from the FCM analysis by Live/Dead dye (BD Biosciences) and SSC-W/SCC-H and FCS-W/FSC-H gating protocols. Dead cells and cells undergoing apoptosis were detected by staining with AnnexinV and 7AAD. All flow cytometry data was acquired on a BD LSR II flow cytometer and analyzed using the FlowJo software package (Tree Star).

### Definition of Cell Types by Flow Cytometry

Cell types were defined by the following markers: T1 B cells (CD19+, IgMhi, IgDlo, CD21-, CD23-) MB B cells (CD19^+^, IgM^hi^, IgD^lo^, CD21^hi^, CD23^lo^) FoB1 cells (CD19+, IgMlo, IgDhi CD21int CD23+), FoB2 cells (CD19+, IgMhi, IgDhi CD21int CD23+) Plasma cells (B220^+^CD138^+^) and Anergic B cells (B220+ AA4+ IgD+ IgMlo). T cells (CD19- CD3+ CD5+), CD4+ T cells (CD19- CD3+ CD4+ CD8-), CD8+ T cells (CD19- CD3+ CD4- CD8+), Tfh cells (CD4+ PD1+ CXCR5+), effector memory T cells (CD44+ CD62L-), Central memory T cells (CD44+CD62L+), Naïve T cells (CD44- CD62L-).

### ^3^H-Thymidine Incorporation Cell Proliferation Assay

Cell proliferation was measured by *^3^*H-Thymidine (Perkin Elmer) incorporation into replicative strands of DNA. Cells were cultured in U bottom microplates and stimulated with the indicated agonists for the specified times either in the continuous presence of 1 microcurie per well of *^3^*H-Thymidine for 48 hours or *^3^*H-Thymidine was added in the last 16 hours of the 72 hours incubation period at 37°C in humidified. The *^3^*H-Thymidine labeled DNA was captured on fiber filter disks, which were then placed in a liquid scintillation counting vials before counting on a scintillation beta-counter.

### Quantitative PCR

RNA was extracted from freshly isolated cells (*ex vivo*) or after culture *in vitro* with agonists using the RNeasy Mini Kit (Qiagen) and used to synthesize cDNA. RNA was quantified on a NanoDrop 1000 prior to use in the RT-PCR reactions. Reverse Transcription was carried out using equivalent amounts of RNA, dNTP, M-MLV reverse transcriptase, RNase inhibitor, nuclease free water, Random Hexamer, 10xPCR buffer and MgCl_2_ (All from Applied Biosystems). Taqman Real time reactions used TaqmanUniversal Master Mix or Taqman Fast Advanced Master Mix (Applied Biosystems) and changes in gene expression were determined by running samples on the Stratagene Max 3000p Detection Systems or Step One Real Time System (Applied Biosystems). Primer/Probe combinations were obtained from applied biosystems; (Mm00477631_m1), Bmf (Mm00506773_m1), Btk (Mm00442712_m1), IFNa1 (Mm03030145_gH), IFNb (Mm00439552_s1), IFNg (Mm00801778_m1), IL-6 (MM00446190), IL12p10 (Mm00434169_m1), Mcl-1 (Mm01257351_g1) MCP-1 (Mm00441242_m1), TNFa (Mm00443258_m1), TLR7 (Mm00446590), and TLR9 (Mm00446193_m1). The relative mRNA fold induction for each gene was calculated relative to 18S ribosomal RNA or GAPDH expression.

### Autoantibody Array

Sera from mice of indicated ages were hybridized to an array containing approximately 75 autoantigens as described (47). Briefly, IgM and IgG antibodies were detected with Cy3 and Cy5 coupled secondary antibodies. Data were normalized to total Ig levels and clustered by antigen. Red indicates greater reactivity than the average for each antigen, green indicates lower reactivity than the average for each antigen.

### ELISA Detection of Antibody Isotypes

Blood samples were obtained by retro-orbital bleeding of mice using heparin containing microcentrifuge tubes. Samples were centrifuged to pellet the RBC before the serum was drawn off and stored at -80 until analysis. For the determination of Igs (total Ig, IgM, IgG1, IgG2a, IgG2c, and IgG3) in the serum/plasma using SBA clonotyping system according to the manufacturer’s instructions (Southern Biotechnology Associates). Briefly, plates were coated with 5 μg/ml of capture Ab, and serum (diluted 1/1,000) was incubated and bound Igs were revealed by HRP-labeled secondary Abs. Results are plotted as the concentration of each Ig isotype.

Serum autoAbs against ANAs, nRNP, SSA, and SSB were measured using ELISA kits (Alpha Diagnostics, San Antonio, TX, USA). Sera were diluted 20-fold before the assay and the manufacturer protocol was followed. Positive values for autoreactive antibodies were determined by the manufacturers cut off value. Soluble BAFF was measured using a mouse BAFF quantikine ELISA respectively (R&D systems). Serum was diluted 50-fold and the manufacturer protocol was followed and ELISA plates were developed and absorbance (absorbance 450nm) using microplate ELISA reader.

### ELISpot for Mouse IgM and IgG

Detection and enumeration of B cells secreting IgM and IgG was determined by ELISpot according to the manufacturer’s instructions. Briefly, antigen was coated onto the ELIspot plate and B cells from the indicated genotypes were incubated on the plate for 16-24 hours before spots were detected. After the incubation time, the plate was washed and biotinylated antigen was added. The plate was washed again prior to the addition diluted Streptavidin-ALP which was incubated for 1 hour. At the completion of the incubation time, the plate was washed and the substrate solution (BCIP/NBT-plus) was added and incubated until distinct spots emerged. The color development was then stopped by washing the plate with tap water and allowed to dry before reading on a ELISpot reader.

### Cytokine Bead Array

Serum cytokine levels were determined by cytokine bead array in accordance with the manufacturer’s guidelines (551287 BD Biosciences). Briefly, serum samples were mixed cytokine labeled capture beads. Each capture bead mixture has a distinct fluorescence when acquired by FCM. The intensity of brightness in the PE channel reveals the cytokine concentration. FCAP Array software (BD Biosciences) was used to calculate results.

### Immunohistochemistry

Spleens, LN, intestines, liver, kidney, salivary glands and other tissues from WT and B*Bim^fl/fl^* mice were formalin (10%) fixed immediately upon harvest, paraffin embedded for H&E staining or frozen in for immunofluorescence staining. Sections were stained with anti- mouse CD19 biotinylated (revealed by streptavidin Alexa Fluor 488) and anti-mouse CD3 (revealed by Alexa Fluor 647 conjugated goat anti-mouse Ab) to visualize B and T cells. Slides were analyzed using a Zeiss Axiovert 200M fluorescence microscope and the Axiovision 4.6 data analysis software program. For H&E staining spleen, kidneys, Pancreas, salivary glands, liver, lungs, and intestines were harvested from control and mutant mice. Tissues were reviewed and scored by a board-certified veterinary pathologist. Scores of inflammation and glomerulonephritis were determined and scored as (0, none; 1, mild; 2, moderate; 3, severe; and 4 extensive). Slides were analyzed and imaged using the Olympus VS120 slide scanner.

### Statistical Analyses

Data collected were compared by two-tailed Students t test. Values of *p ≤ 0.05 were considered statistically significant.

## Results

### B Cell-Specific Deletion of Bcl2l11 (BBim^fl/fl^) Leads to Expansion of B and T Cells

**Figure 1:**
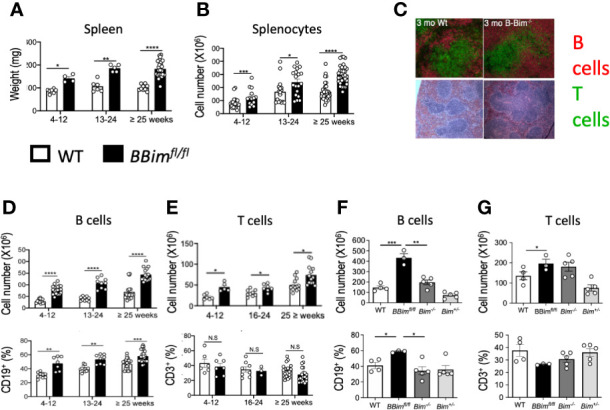
B cell lineage-specific deletion of Bim resulted in expansion of splenic B and T cells. Spleen weights and splenocyte numbers, B cells and T cells were identified by FCM using established cell surface markers as described in materials and methods. **(A)** Spleen weights at different ages from *BBim^fl/fl^ and* WT mice. **(B)** Total splenocyte numbers. **(C)** Immunofluorescence image showing splenic follicles (top panel) stained for B220+ B cells (red) and CD3+ T cells (green) and H&E images (bottom panel) showing follicular structure and lymphocyte organization (top panel) of spleens from 3 month old mice. **(D)** Numbers (top panel) and proportions (bottom panel) of splenic **(D)** B cells and **(E)** T cells. **(F, G)** Quantification of splenic B and T cells in *BBim^fl/fl^* relative to *Bim^-/-^* mice. Numbers (top panel) and proportions (bottom panel) of **(F)** B cells and **(G)** T cells. Data is representative of >3 independent experiments. Not significant (N.S) P > 0.05, *P ≤ 0.05, **P ≤ 0.01, ***P ≤ 0.001, ****P ≤ 0.0001, calculated by Students T- test and Mann-Whitney non-parametric test.

To address the significance of B cell-intrinsic regulation of apoptosis in establishing tolerance in the wild type B cell repertoire, we crossed a CD19-cre mouse to a mouse carrying Loxp flanked Bim gene (Bcl2l11) alleles in C57BL/6 background, previously described by Korsmeyer group (43). The mouse produced by this intercross was confirmed for Bim deletion in the B lineage, termed *BBim^fl/fl^* (**Supplementary Figure 1A**). We found that *BBim^fl/fl^* mice developed splenomegaly and lymphadenopathy at a relatively early age with a corresponding increase in splenic weight, size and cellularity (**Figures 1A, B** and **Supplementary Figure 2A**). Splenic lymphoid follicles appeared normal in young *BBim^fl/fl^* mice (**Figure 1C**), but with age the lymphoid follicles in the *BBim^fl/fl^* mice were enlarged and fused due to enlarged B cell zones and became disorganized in one year and older mice (**Supplementary Figure 2D**). The overall splenic B cell numbers were increased 2-3 fold in young adult *BBim^fl/fl^* mice relative to littermate control mice (**Figure 1D**), consistent with recent reports of B cell-specific Bim deletion (39, 48). In contrast to the previous reports, the increase in B cell numbers and ratios was maintained into relatively old age (**Figure 1D**).

The overall T cells were also variably increased in the *BBim^fl/fl^* relative to control mice but to a lesser extent relative to the expansion of B cells (**Figure 1E**). Like B cells, the increases in T cells did not wane with age (**Figure 1E**).

Prior studies have reported that the SLE-like phenotype observed in systemically Bim-deficient mice (*Bim^-/-^*) in a mixed genetic background (SV129 x C57BL/6) was much milder in pure C57BL/6 background (35, 36, 49). Therefore, we compared overall B and T cell proportions and numbers in the *BBim^fl/fl^* mice with *Bim^-/-^* mice, both in C57BL/6 background. In agreement with the prior reports, we found that the numbers of B cells were increased (1.5-2-fold) in *Bim^-/-^* mice relative to WT. However, the expansion of B cells in the *BBim^fl/fl^* mice was greater (3-fold) relative to *Bim^-/-^* mice (**Figure 1F**), whereas T cells were increased comparably (1.5-2-fold) between the two genotypes (**Figure 1G**). These data suggest a critical B cell-autonomous role for Bim in B cell homeostasis, which is more apparent in the Bim-sufficient milieu in the *BBim^fl/fl^* mice. In addition, Bim-sufficient T cell compartment also appears to be influenced by dysregulated B cells directly and/or indirectly including an increase in T cells.

**Figure 2:**
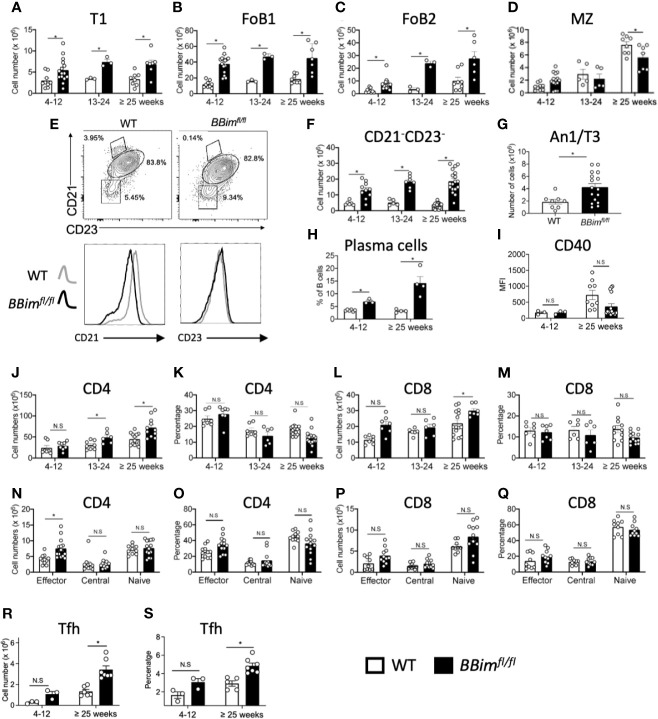
*BBim^fl/fl^* mice have expanded effector B and T cell populations in the spleen. Splenic B and T cell subpopulations were identified by FCM using established cell surface markers described in materials and methods. **(A–D)** Numbers of T1, FoB1, FoB2 and MZ plus pre-MZ B cells subsets in *BBim^fl/fl^* and WT mice in the indicated age groups. **(E)** Representative FCM plots showing gating strategy to quantify CD21 and CD23 double negative (DN) B cells within total B cells (top panels) and histograms displaying CD21 and CD23 expression showing reduced CD21 expression in B cells from *BBim^fl/f^* ^l^ relative to WT mice (bottom panels). **(F)** Numbers of CD21^-^ CD23^-^ DN B cells in *BBim^fl/fl^* and WT mice. **(G)** Numbers of anergic (An1/T3) B cells. **(H)** Numbers of plasma cells. **(I)** MFI values of CD40 in B cells **(J–Q)** Quantification of splenic CD4 and CD8 T cell populations on CD3+ gated cells in the ≥ 25 week old *BBim^fl/fl^* and WT mice. Representative graphs displaying **(J)** CD4 T cell numbers and **(K)** proportions and **(L)** CD8 T cell numbers and **(M)** proportions. **(N)** Cell numbers and **(O)** proportions of CD4 effector, central memory and naïve CD4 subsets. **(P)** CD8 T cell numbers and **(Q)** proportions of CD8 effector, central memory and naïve T cells. **(R)** Cell numbers and **(S)** percentages of Tfh cells. Data is representative of >3 independent experiments. Not significant (N.S) P > 0.05, *P ≤ 0.05, calculated by Students T- test and Mann-Whitney non-parametric test.

In young *BBim^fl/fl^ mice* (4-12 wk old) the numbers of all mature FoB1 and FoB2 and T1 B cell subsets were increased 2-4 fold, except B1 B cells (**Figures 2A–C** and data not shown). In contrast, the proportions of MZ and precursor (pMZ) B cells were significantly decreased in *BBim^fl/fl^* mice with age relative to WT mice, which also reflected in a modest decrease in their numbers (**Figures 2D, E**). In what may be a related finding, the reduction in CD21 expression was greater in *BBim^fl/fl^* than WT B cells (**Figure 2E**, bottom panels). We noted that CD21^-^ and CD23^-^ double negative (DN) B cells were significantly increased in *BBim^fl/fl^* spleens and progressively further increased with age relative to WT (**Figure 2F**). Given increased numbers of T1 B cells escape deletion in the *BBim^fl/f^*, we wondered whether anergic (An1/T3) B cells that have a shorter life span and are hypersensitive to apoptosis may live longer and accumulate, consistent with previous findings (34). Indeed. the anergic An1/T3 B cells increased in the *BBim^fl/fl^* mice (**Figure 2G**). The abnormal increases in T1 and An1/T3 B cells may be consequential for autoAb production as plasma B cells were also increased (**Figure 2H**). An increase in T1 B cells, which serves as a first peripheral tolerance checkpoint suggests that this otherwise apoptosis-sensitive population survives negative selection. Likewise, An1 B cells also acquire resistance to apoptosis and may contribute to break in tolerance in *BBim^fl/fl^* mice. The potential for T-B interaction was investigated by evaluating CD40 on B cell and helper T cells. The cell surface CD40 expression is reduced in lupus patient B cells that resemble ABC B cells (50), but there was no difference in the CD40 levels in WT and in *BBim^fl/fl^* B cells (**Figure 2I**). The CD4 and CD8 T cell numbers were increased with age but proportions were not significantly altered (**Figures 2J–M**). The T cell subset analysis revealed that effector CD4 T cells were increased (**Figures 2N, O**), but CD8 T cells were not (**Figures 2P, Q**). These data suggest that dysregulated apoptosis in B cells by loss of Bim promotes accumulation of autoreactive B cell populations and CD21^lo^CD23^lo^ B cells, which may be related to SLE-associated Tbet^+^ B cells (51), along with an increase in CD4 T effector and Tfh cells (**Figures 2R, S**) raise the possibility of autoimmune pathogenesis in *BBim^fl/fl^* mice. This potential is also supported by the findings that *BBim^fl/fl^* mice die earlier than their WT counterparts (**Supplementary Figure 2E**).

### *BBim^fl/fl^* Mice Display Systemic Autoimmunity

**Figure 3:**
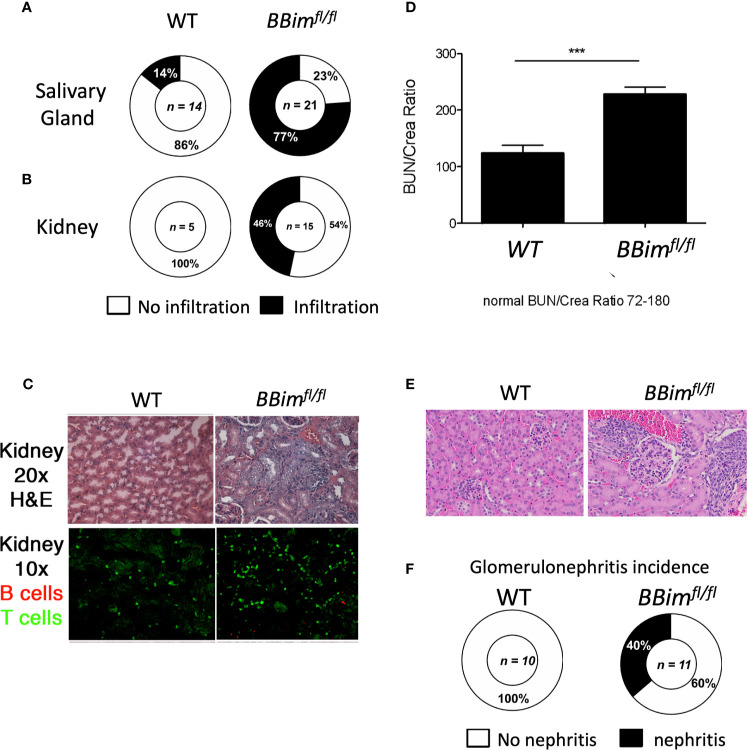
*BBim^fl/fl^* mice exhibit multiorgan lymphocyte infiltration and kidney damage. Tissues from ≥ 6 month old WT (n ≥3) and *BBim^fl/fl^* (n ≥8) mice were assessed for tissue damage. Representative pie charts show incidence of inflammation in the **(A)** salivary glands, and **(B)** kidneys. **(C)** Representative H&E images showing glomeruli in kidney sections (top panels) and immunofluorescence images of kidney sections (bottom panels) showing infiltrated T cells (green) and B cells (red) **(D)** Blood urea nitrogen and plasma creatine levels (BUN/crea) in the blood (age ≥ 6 month old) **(E)** H&E stained images of kidney sections showing enlarged glomeruli (glomerulonephritis) in *BBim^fl/fl^* relative to WT mice. **(F)** Incidence of kidney damage in the *BBim^fl/fl^* and WT mice. ***P ≤0.001, calculated by Students T-test.

Our data suggests that Bim mediated apoptosis is critical for B cell homeostasis and loss of this regulatory function may lead to autoimmune pathogenesis, which is not consistent with previous reports (39, 40, 52). We therefore, aimed to better define the pathophysiological effects of B cell specific loss of Bim, we analyzed various tissues for immune cell infiltration and damage in cohorts of *BBim^fl/fl^* and WT mice at different ages. We found that with age (6 months and older), the *BBim^fl/fl^* mice displayed lymphocytic infiltration in the liver and the lungs (**Supplementary Figure 2B**), as previously described for *Bim^-/-^* mice (35, 43). In addition, the majority of the *BBim^fl/fl^* mice had immune cell infiltration in the SGs, and kidneys (**Figures 3A–C** and **Supplementary Figure 2C**). Given these tissues are affected in SLE and SS they were further analyzed by immunohistochemistry. We found that glomeruli in *BBim^fl/fl^* mice were disorganized and damaged (**Figure 3C** upper panels) with evidence of T cell infiltration and to a lesser extent B cell infiltration, (**Figure 3C** lower panels). Consistently, *BBim^fl/fl^* mice also showed an increase in Bun/Crea ratio in the blood (**Figure 3D**). The glomerular damage was present in 40% of *BBim^fl/fl^* mice but not observed in the WT controls (**Figures 3E, F**).

**Figure 4:**
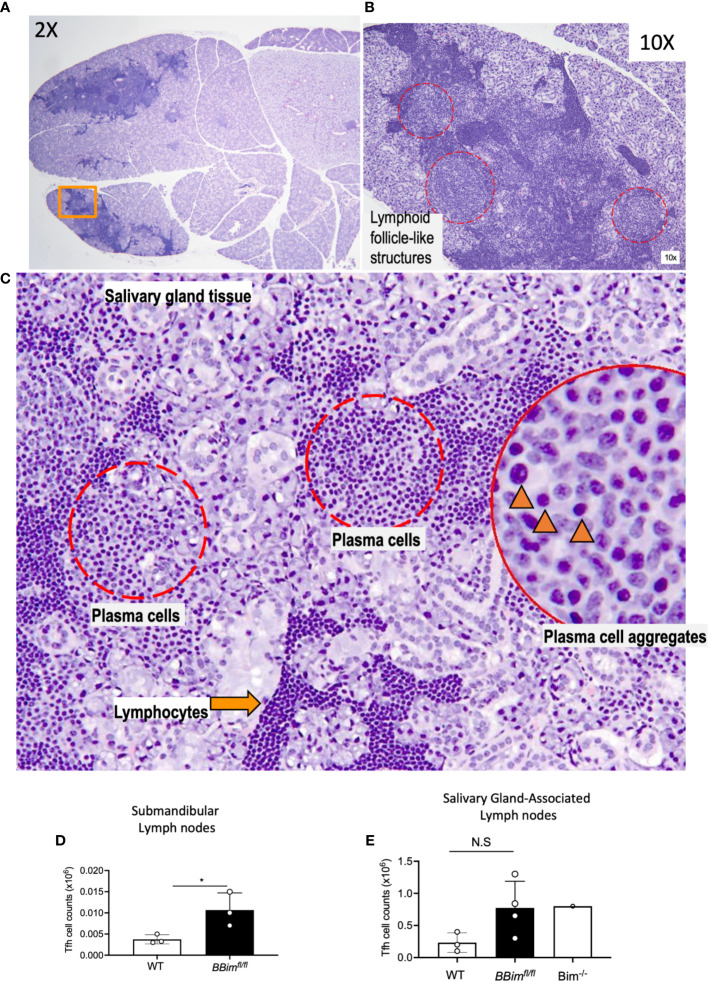
Salivary glands in *BBim^fl/fl^* mice display ongoing immune reaction. **(A)** Salivary gland from an 8.5 month old *BBim^fl/fl^* mouse showing aggregates of lymphoid infiltrates (H&E, 2x magnification). **(B)** Region of salivary gland indicated by an orange rectangle in **(A)**, showing pale areas with of lymphoid follicle-like structures (red dotted circles, 10x). **(C)** Higher magnification (20x) of one of these areas demonstrates plasma cell aggregates (red dotted circles); plasma cells are identified by ovoid cells with abundant pale basophilic cytoplasm and eccentric nucleus (inset, black arrowheads, 40x), adjacent to lymphocytic infiltrates identified by smaller cells with scant cytoplasm and prominent dark round nucleus (orange arrow). **(D)** Numbers of Tfh cells in the submandibular lymph nodes and **(E)** salivary gland associated lymph nodes. Not significant (N.S) P > 0.05, *P ≤ 0.05, calculated by Students T- test.

Enlarged SGs are among the characteristic features of SS, therefore, *BBim^fl/fl^* SGs were more closely examined by histology and expanded to immune cell phenotype by FCM. Histological examination of the SGs revealed significant lymphoid hyperplasia, consisting of small lymphocytes (**Figure 4A**). Furthermore, immune cell infiltrates in the SGs formed follicle-like structures populated with B cells and plasma cells (PCs) (**Figures 4B, C**). These data suggest chronic inflammation, which may result in the loss of SG acini and impaired SG function. To uncover any T cell contribution to the PC formation FCM analysis of submandibular LNs was performed. The results revealed a two-fold increase in the T follicular helper cells (PD1^+^CXCR5^+^Bcl6^+^) in *BBim^fl/fl^* relative to WT mice (**Figures 4D, E** and **Supplementary Figures 3A–D**). Tertiary lymphoid structures (TLSs) are often formed at the site of inflammation and are associated with autoimmune inflammation (8, 53). We found that *BBim^fl/fl^* mice had TLSs in the proximity of SGs as well as in the abdomen as a network of strings of lymph node-like structures (**Supplementary Figure 4A**). The TLS in the *BBim^fl/fl^* mice were composed of different proportions of B cells, T cells and dendritic cells in different mice. B cell phenotype was also distinct containing different ratios of CD23^-^CD21^+^ MZ-like and CD21^+^CD23^+^ FoB like B cells (**Supplementary Figure 4B**). The extent of infiltration and neogenesis of TLS varied between mice. Taken together, these data support the notion that apoptosis-resistant B cells escape self-tolerance mechanisms in *BBim^fl/fl^* mice and can mediate SS- and SLE-like autoimmune pathology. Our findings differ with recent studies reporting no obvious autoimmune pathology in mice with B cell-specific Bim deletion, perhaps due to analysis limited to relatively young mice (39, 48).

### SLE and Sjögen’s Signature autoAbs in *BBim^fl/fl^* Mice

**Figure 5:**
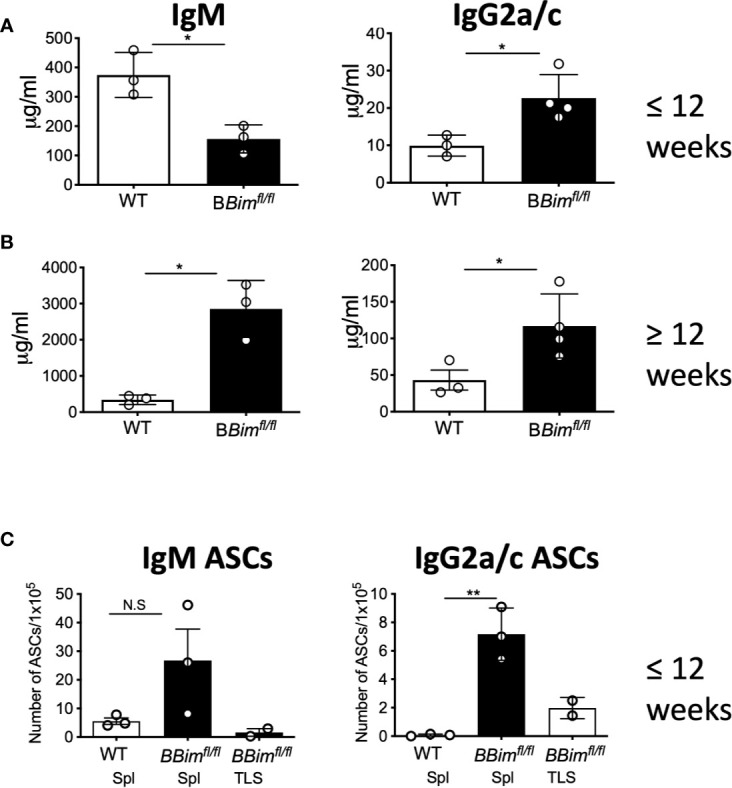
*BBim^fl/fl^* mice exhibit hypergammaglobulinemia and increased ASCs. Quantification of serum immunoglobulins and ASCs in WT and *BBim^fl/fl^*mice. **(A)** IgM and IgG2a/c levels in serum from mice ≤ 12 weeks old **(B)** IgM and IgG2a/c levels in mice ≥ 12 weeks old mice. Numbers of **(C)** IgM and IgG2a/c ASCs in splenocytes in mice ≥ 12 weeks old. Data is representative of >3 independent experiments. Not significant (N.S) P > 0.05, *P ≤ 0.05, **P ≤ 0.01, calculated by Students T-test.

To extend our findings of systemic autoimmunity in *BBim^fl/fl^* mice, serum immunoglobulins were measured by ELISA. While both young and old *BBim^fl/fl^* mice displayed increased circulating IgG2a/c (**Figures 5A, B**, right panels). The IgM levels were low in young mice but increased in older mice (**Figures 5A, B**, left panels), whereas IgG1 increased only in the young mice and IgG3 was not changed (**Supplementary Figures 5A, B**). Consistently, IgG2a/c antibody secreting cells (ASCs) were also increased in the older *BBim^fl/fl^* mice (**Figure 5C**).

**Figure 6:**
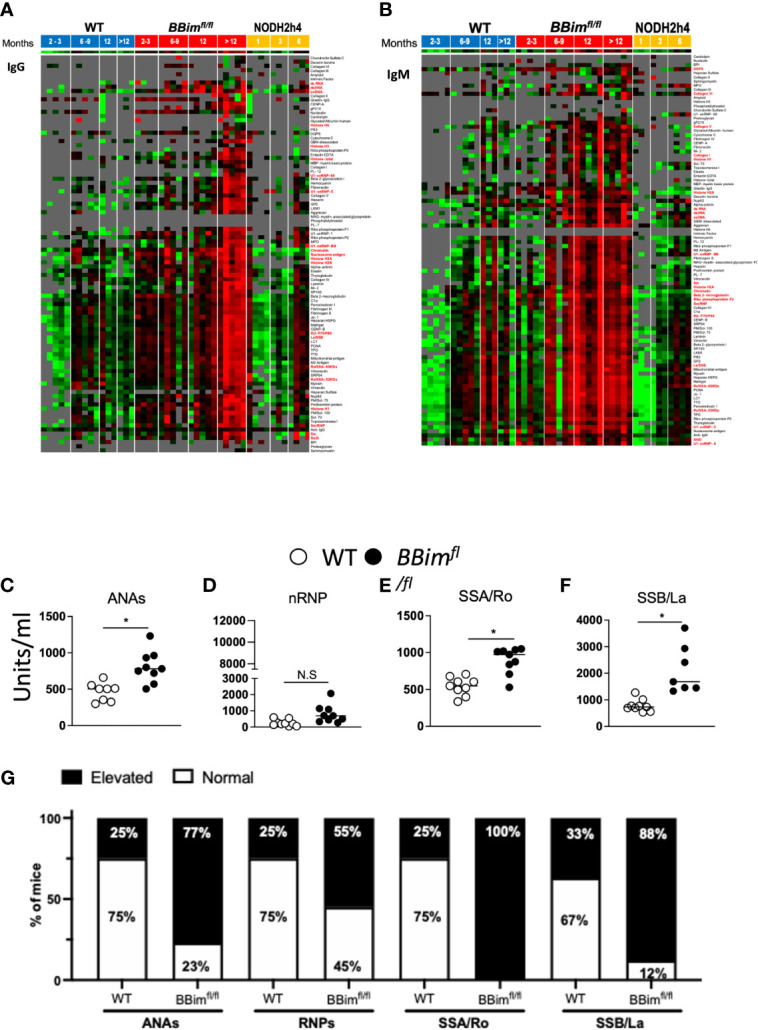
*BBim^fl/fl^* mice exhibit elevated titers of SLE/SS signature autoAbs. Serum from mice of indicated age groups were screened for autoAbs by hybridizing to an array containing over 90 autoantigens. Each serum specimen was taken from a different mouse so each time point would be independent. Red boxes indicate greater reactivity than the average for each antigen and green boxes indicate lower reactivity than the average for each antigen. Reactivities close to the mean are displayed in black/gray. Some of the SS and SLE-associated autoAb names are highlighted in red. **(A, B)** Heat maps with clustering of WT (left), *BBim^fl/fl^* (middle) and NODH2h4 (right). Supervised clustering of autoAbs was performed with normalized signal intensities for baseline IgG and IgM autoAbs. **(A)** IgG and **(B)** IgM autoAbs showing higher reactivity (red) appeared in serum of *BBim^fl/fl^* mice by 6-9 months. At 12 months, additional IgM antibodies emerged and persisted, which were again followed by the subsequent development of IgG antibodies in most instances. In each case tested (with the exception of PR3 antibodies (vasculitis-associated) which remained persistently IgM only), IgM antibodies appeared at time points either concurrent with or prior to the emergence of IgG antibodies, and both IgM and IgG persisted through all subsequent time points. The autoantibodies that emerged included all of the tested SS-associated antibodies (Ro52/SS-A, Ro60/SS-A, La/SS-B, CENP-B), and a variety of lupus-associated autoantibodies (chromatin, ssDNA, alpha-actinin, vitronectin, snRNP, Beta2-GPI, PCNA, nucleosome Ag, Sm-D, Histone H2B, peroxiredoxin 1, ribophosphoprotein P0, myosin, Heparan HSPG, Matrigel, Vitronectin, Heparin, collagen IV). Additional autoantibodies emerging in the same time frame included those targeting some autoimmune hepatitis antigens (LKM1, mitochondrial Ag, LC-1), some myositis-associated antigens (SRP54, Jo-1, Nup6.2), celiac disease –associated targets (DGPS, TTG), as well as the following antigens: C1alpha, the Crohn’s-associated antigen GP2, and the thyroiditis-associated antigen TPO. At 12 months, additional IgM antibodies emerged and persisted, followed by the development of IgG in most instances includingU1-snRNP-BB, U1-RNP-C, laminin, hemocyanin, aggrecan, fibrinogen s, autoimmune hepatitis antigens (M2 antigen, SP100), and a scleroderma/myositis-associated target (PM-Scl75). However, some of IgM autoAbs were not accompanied/followed by IgG autoAbs, exemplified by the myositis –associated antigen PL12 and neuropathy-associated myelin associated glycoprotein-Fc (MAG) and collagen V (in older mice).In addition, aged BBimfl/fl mice developed some additional IgM autoAbs to lupus-associated autoantigens in the majority of tested mice including GBM-associated, and prothrombin protein, which were associated with concurrent emergence of IgG against the same targets. Several autoAbs were upregulated only in aged mice and only as IgG, including additional lupus-associated specificities (U1-snRNP68, U1-snRNP1, Sm, total histone, histone H2A, Histone H1, Heparan Sulfate, Entactin-EDTA, collagen II, fibronectin, elastin, fibrinogen IV, ribophosphoprotein P1, ribophosphoprotein p2), a myositis-associated target (Mi-2), some scleroderma-associated specificities (CENP A, Ku P70-p80, Scl-70, Topoisomerase I, PM-Scl100), an autoimmune hepatitis-associated target (gp210), a thyroiditis-associated antigen (thyroglobulin), and the vasculitis-associated target MPO. **(C–F)** Serum from*BBim^fl/fl^ and WT* mice (age ≥ 24 weeks old) were assessed for autoAbs distinctive for SS and SLE by ELISA. **(G)** Representative bar graphs show the frequency of elevated (filled bar) and normal (open bar) autoantigens titers in WT and *BBim^fl/fl^* mice. Elevated titers were determined by calculating the positive index as stated in the ELISA kit protocol (alpha diagnostic international). Data is representative of >3 independent experiments. Not significant (N.S) P > 0.05, *P ≤ 0.05, calculated by Students T-test and non-parametric Mann-Whitney test.

For a comprehensive analysis of autoAb breadth and specificity, we used an autoantigen (autoAg) array representing more than 90 of the most common autoantigens in serum from mice grouped by ages; 2-3, 6-9 months, one-year and over one-year. IgM and/or IgG autoAbs to several known autoantigens particularly those characteristic of SLE and SS were present in the serum from mice of the indicated genotypes and ages (**Figures 6A, B**). Each serum specimen was taken from a different mouse so each time point would be independent. Many autoAbs showed overexpression in the majority of *BBim^fl/fl^* mice, whereas this pattern was not seen with any autoantibodies at any time points for the WT or NODH2h4 mice. Among the earliest autoAbs to emerge were all of the tested SS-associated antibodies (Ro52/SS-A, Ro60/SS-A, La/SS-B, CENP-B), and a variety of lupus-associated autoantibodies targeting DNA complexes, ribonucleoproteins, and connective tissue/structural antigens, collectively including nuclear, cytoplasmic, membrane-bound, and extracellular targets. Over time, many additional lupus-associated autoantibodies emerged. All the SS and SLE autoantibodies showed persistence over time, and showed progression from IgM to also IgG expression (**Figures 6A, B**).

The presence of autoAbs to autoantigens characteristic of SS and SLE including anti-dsDNA, -sm/RNP, -La/SSB, -Ro/SSA were confirmed by ELISA (**Figures 6C–G**). Some of older *BBim^fl/fl^* mice had a tendency of increased autoreactive ASCs in the LNs associated with the sSGs relative to WT controls, although only anti-SSA IgM was statistically significant (**Supplementary Figure 6**). Together, autoAb array, ELISA and ELISpot data demonstrate that SLE/SS characteristic autoAbs were elevated much more frequently among *BBim^fl/fl^* mice relative to WT controls.

**Figure 7:**
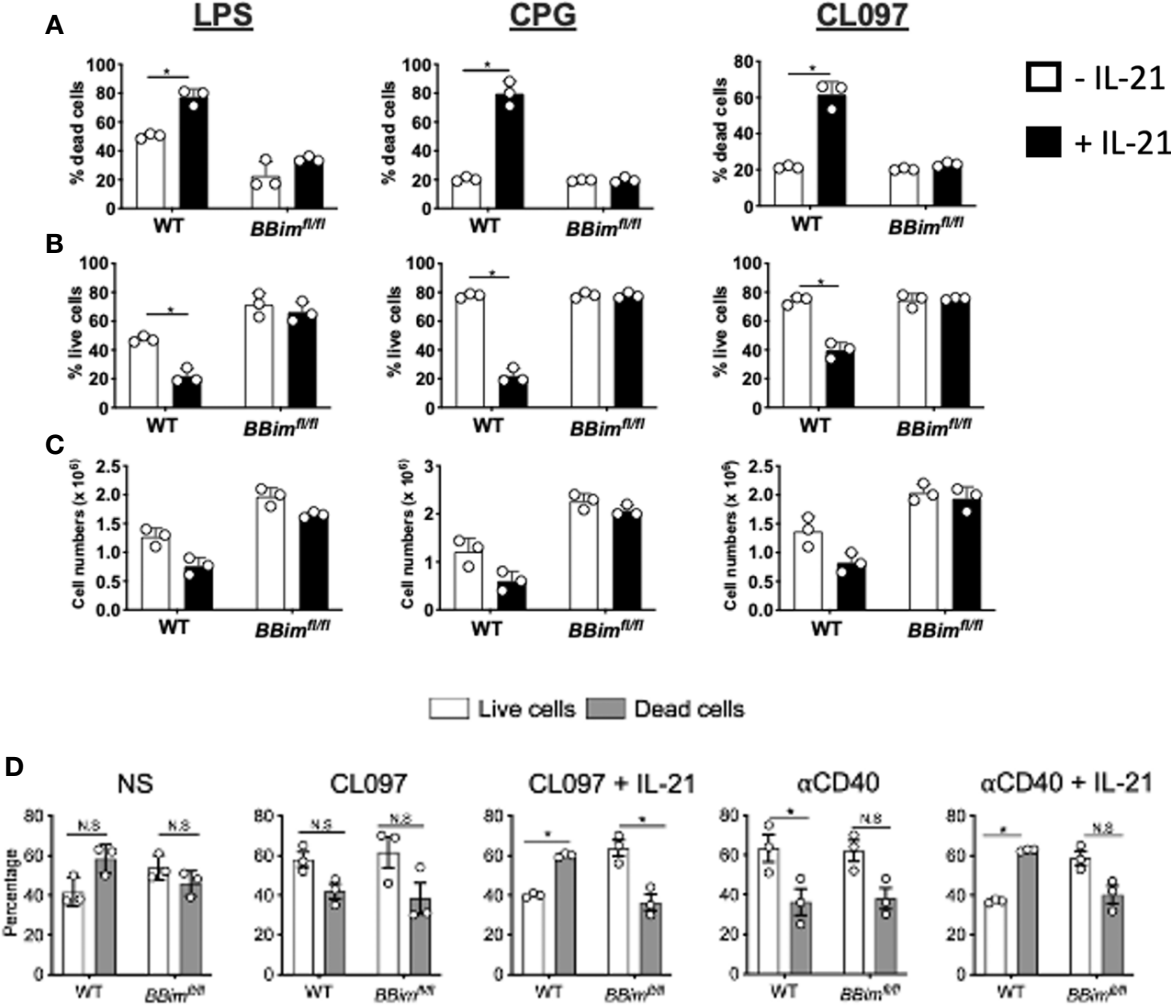
*BBim^fl/fl^* B cells are resistant apoptosis costimulated with IL-21 and *via* TLR4, TLR7, TLR9 or CD40. Purified B cells from WT and *BBim^fl/fl^* mice were incubated for 48 hours with the indicated TLR agonists or anti-CD40 in the absence or in the presence of IL-21 (25ng/ml) and then analyzed for **(A)** dead cells and **(B)** live cells distinguished using a fixable viability dye (Invitrogen). **(C)** Numbers of live B cells in the cultures (trypan blue-) **(D)** Splenocytes from *BBim^fl/fl^* and WT mice were treated with TLR7 agonist (CL097) or anti-CD40 Abs in the absence or in the presence of IL-21 (25ng/ml), stained with annexin V and 7AAD and analyzed by FCM. Bar graphs showing percentages of B cells that are live (white; 7AAD- Annexin V-) and dead (gray; 7AAD+/- Annexin V+) after culture for 48 hours. Not significant (N.S) P > 0.05, *P ≤ 0.05, calculated by Students T-test.

Dysregulation of B cells and autoAb production in SLE-like autoimmune disease are influenced by both innate (TLR) and adaptive (CD40) pathways and Tfh secreted IL-21 which regulates B cell differentiation into plasma cells, memory B cells and CD11c^hi^ ABC B cells (50, 54, 55). IL-21 can regulate B cell proliferation, differentiation or apoptosis in a context-dependent manner (56, 57). For example, IL-21 can promote both proliferation and differentiation as well as apoptosis in B cells costimulated with anti-CD40, whereas this combination can rescue BCR and TLR9 induced cell death (23, 55–57). Mechanistically, IL-21 inhibits TLR4 and TLR9 induced proliferation by downregulating anti-apoptosis members of the Bcl-2 family and inducing Bim-dependent apoptosis (23, 55–57). Due to relevance of both adaptive and innate pathways in autoAb production (58, 59), we determined whether B cells in the *BBim^fl/fl^* mouse model are protected from IL-21 induced apoptosis under TLR or CD40 costimulatory conditions (**Figures 7A–D** and **Supplementary Figures 7A–C**). The *BBim^fl/fl^* B cells were more resistant to IL-21-induced apoptosis relative to WT controls in response to stimulation *via* TLR4 (LPS), TLR9 (CpG),TLR7 (CL097), and anti-CD40 Abs (**Figures 7A–D** and **Supplementary Figure 7A**). Although, the anti-CD40 Abs enhanced B cell viability similarly in both WT and *BBim^fl/fl^* B cells, IL-21 induced significant apoptosis in WT B cells but not *BBim^fl/fl^* B cells (**Figure 7D** and **Supplementary Figure 7A**), as previously shown (56). Similar results were obtained with costimulation of peripheral blood B cells with IL-21 plus CL097 and IL-21 plus anti-CD40 (**Supplementary Figure 7B, C**). The rescue of IL-21 induced apoptosis by CD40 may require use of optimal dose and presentation of CD40L (e.g., membrane bound CD40L) as was recently reported (55). These data suggest that B cells from *BBim^fl/fl^* mice are significantly protected from IL-21 induced apoptosis under both innate and adaptive costimulatory conditions and that innate, adaptive or both mechanisms may contribute to autoAb production targeting autoantigens associated SS and SLE in *BBim^fl/fl^* mice.

### *BBim^fl/fl^* B Cells Can Proliferate Upon BCR and TLR Stimulation

**Figure 8:**
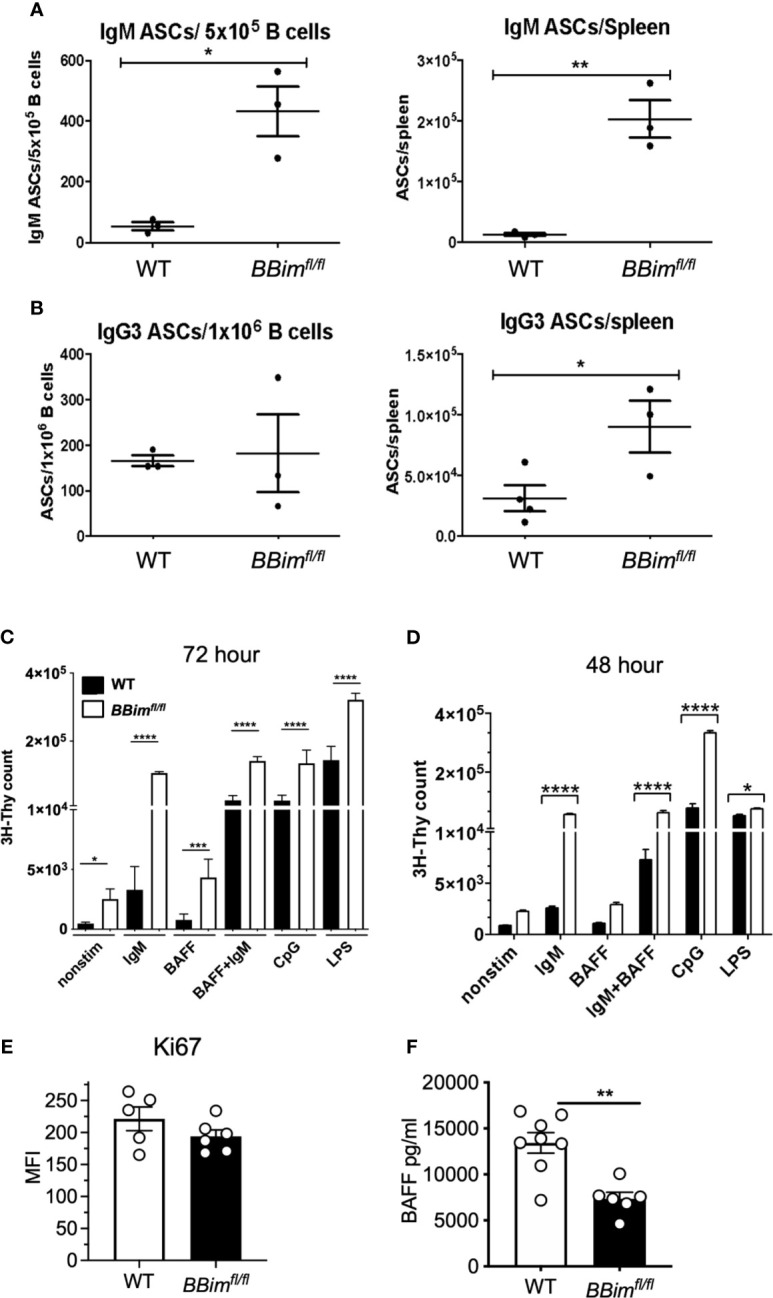
BCR and TLR induced *BBim^fl/fl^* B cell proliferation is enhanced by BAFF. B cells from WT and *BBim^fl/fl^* mice were analyzed for differences in activation and proliferation. WT and *BBim^fl/fl^* mice were immunized with TNP-Ficoll, before measuring B cell and antibody response by ELISpot. Quantification of **(A)** IgM and **(B)** IgG3 antibody secreting B cells from *BBim^fl/fl^* and WT mice. **(C)** *^3^*H-Thymidine incorporation assay after treatment with indicated agonist for72 hours. **(D)** *^3^*H-Thymidine incorporation of B cells treated with the indicated agonist for 48 hours. **(E)** MFI of Ki67 in B cells from *BBim^fl/fl^* and WT mice. **(F)** Circulating BAFF levels in serum from *BBim^fl/fl^* and WT mice. *P ≤ 0.05, **P ≤ 0.01, ****P ≤ 0.0001, calculated by Students T-test.

To understand the underpinnings of autoimmunity in *BBim^fl/fl^* mice, B cells were analyzed for spontaneous and induced survival and proliferation. Comparable expression of Ki67 in B cells isolated from WT and *BBim^fl/fl^* mice indicated that Bim-deficiency does not impair B cell proliferation *in vivo* (**Figure 8E**). To assess BCR induced proliferation *in vivo*, we immunized mice with TNP-Ficoll, a prototypical T cell-independent type-2 antigen that induces clonal B cell expansion and production of antibody response without T cell help (**Figures 8A, B**). The data demonstrated that the numbers of IgM and IgG3 ASCs were greater in the *BBim^fl/fl^* than in the WT control mice. These results demonstrate that B cells in *BBim^fl/fl^* mice are capable of proliferation *in vivo* and can do so upon engagement of the BCR with antigen.

Because altered homeostasis of the B cell compartment is likely the driving force in the development of autoimmunity in *BBim^fl/fl^* mice, we sought to determine mechanisms of B cell expansion. Therefore, the ability of *BBim^fl/fl^* B cells to proliferate *in vitro* was assessed in response to stimulation *via* TLR4, TLR9 and the BCR with or without combination with BAFF. Purified B cells were cultured in the presence or absence of anti-IgM (Fab_2_), BAFF, LPS, and CpG individually or in the indicated combinations for 72 hrs or for a shorter duration (48h) and B cell proliferation measured by DNA synthesis by ^3^H-Thymidine incorporation (**Figures 8C, D**). The results showed that *BBim^fl/fl^* B cells proliferated more than the WT controls and BAFF enhanced BCR stimulatory effect, suggesting that *BBim^fl/fl^* B cells can respond to stimulation *via* key receptors that regulate B cell survival and proliferation. A greater effect of BAFF on BCR induced proliferation of the mutant compared to WT cells could result from *in vivo* BAFF deprivation as indicated by reduced circulating BAFF in *BBim^fl/fl^* mice (**Figure 8F**). Overall, our data shows that Bim-deficient B cells isolated from *BBim^fl/fl^* mice can proliferate *in vivo* and can be induced to proliferate *in vitro*. These results are consistent with prior studies showing that Bim-deficient B cells can proliferate normally (43), whereas they differ with another study reporting defective proliferation (60). The reasons for these differences are not clear as both prior studies used *Bim-/-* mice generated in the Korsmeyer laboratory (43). In fact, our data indicates that B cells isolated from *BBim^fl/fl^* mice can proliferate better under certain conditions, possibly due to the differences in the *in vivo* milieu in mice with B cell-specific deletion compared to the mice lacking Bim in all cells, as indicated by gene expression differences in B cells from the two strains (**Supplementary Figure 8**). An alternative possibility is that the proportion of *BBim^fl/fl^* B cells that undergo cell division is reduced relative to WT, but their numbers are increased due to resistance to BCR and TLR activation induced cell death in cultures (61, 62). These *BBim^fl/fl^* B cells undergo DNA synthesis and incorporate ^3^H-Thymidine independent of the history of cell cycle and number of cell divisions. Consistent with this interpretation, prior studies have shown that while there are substantial numbers of non-dividing cells in Bim-deleted B cell cultures, the number of B cells that had undergone cell division was not significantly different relative to the WT B cells (40). Together, these results suggest that at least a proportion of B cells from *BBim^fl/fl^* mice undergo cell division and BAFF may also contribute to B cell expansion in addition to prolonged survival.

To further dissect the underlying reasons for the differences in proliferation of B cells in *BBim^fl/fl^* mice and those isolated from *Bim^-/-^* mice previously reported, we compared gene expression profiles between the two genotypes. This analysis included genes that may display compensatory expression such as Bim-related genes encoding BH-3 only pro-apoptotic, along with the anti-apoptotic genes of the Bcl-2 family as well as TLRs and the TLR and BCR signal mediator, Btk, which has been implicated in B cell mediated autoimmunity in mice (63). RNA from purified B cells isolated from *Bim^-/-^*, *Bim^-/+^*, *BBim^fl/fl^* and WT mice was subjected to qRT-PCR. We found that Bmf mRNA was increased in *BBim^fl/fl^* B cells (3-4 fold) relative to those isolated from Bim^-/-^, Bim^-/+^ and WT mice (**Supplementary Figure 8A**). The expression of Blcl2 or Mcl1 was reduced in *Bim^-/-^* B cells relative to WT and *B-Bim^fl/fl^* B cells (**Supplementary Figures 8C, D**). These results suggest differences in gene expression profiles in B cells lacking Bim based on whether they developed in a Bim-deficient *versus* Bim-sufficient milieu. Increased BH-3 only pro-apoptotic Bmf, which functions in a similar manner to Bim in B cells as deletion of both genes compounds the increase in B cell phenotype (52), suggest some functional compensation of loss of Bim in the *BBim^fl/fl^* B cells. Interestingly, Btk mRNA was also modestly increased in the *BBim^fl/fl^* B cells relative to *Bim^-/-^* B cells (**Supplementary Figure 8B**). In contrast, TLR7 and TLR9 mRNA were largely comparable among the genotypes (**Supplementary Figures 8E, F**), suggesting altered TLR expression is not responsible for increased proliferation *via* TLR7 or TLR9 (**Figures 8C, D**). In this context, both Btk and BH3 only proteins are involved in regulating intracellular Ca2^+^ flux that is important for B cell proliferation. Thus, an increase in Bmf and Btk may contribute to restoring *BBim^fl/fl^* B cell proliferation relative to B cells from Bim^-/-^ mice. The nature of this compensation and the inductive signals that drive Bmf and Btk expression remain to be elucidated by careful comparative studies.

### Deletion of Btk in *BBim^fl/fl^* Mice Reduced Symptoms of Autoimmunity

**Figure 9:**
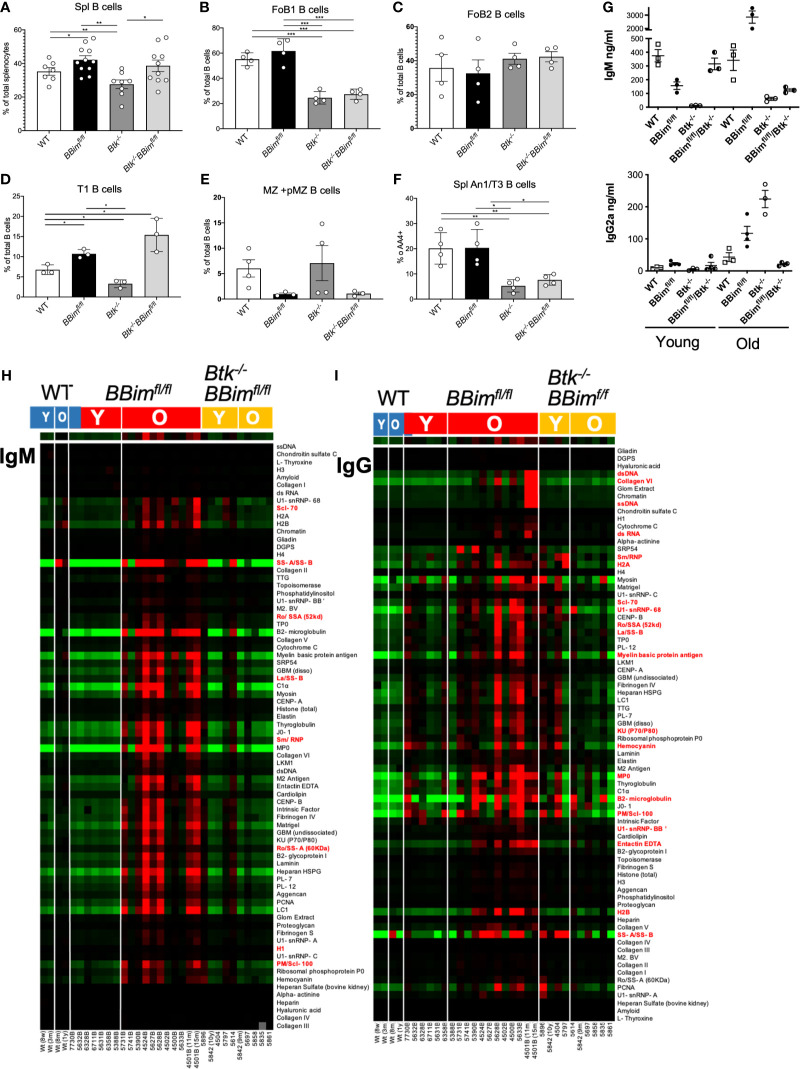
Deletion of Btk in *BBim^fl/fl^* mice reduced symptoms of autoimmunity. **(A)** Graph displaying splenic B cell percentages in WT, *BBim^fl/fl^*, *Btk^-/-^* and *Btk^-/-^BBim^fl/fl^* mice. **(B–F)** Representative graphs comparing percentages of the listed B cells subpopulations in WT, *BBim^fl/fl^*, Btk^-/-^, and *Btk^-/-^ BBim^fl/fl^* mice. **(G)** Basal IgM and switched IgG2a antibodies in the serum of young (≤ 12 weeks old) and old (≥ 12 weeks old) mice. **(H, I)** Heat map with clustering of autoAbs in the serum from WT (left), *BBim^fl/fl^* (middle) and *Btk^-/-^BBim^fl/fl^* (right) mice of indicated ages **(H)** IgM and **(I)** IgG autoAbs. *P ≤ 0.05, **P ≤ 0.01, ***P ≤ 0.001, calculated by Students T-test.

BCR and TLR signaling control B cell selection, growth, activation and differentiation into antibody secreting cells (ASCs). There is a modest increase in Btk gene expression in *BBim^fl/fl^* mice (**Supplementary Figure 8**) and B cells from *BBim^fl/fl^* mice express increased levels of certain cytokines (IL-6, IL-10 and IFNa) in response to stimulation *via* TLR7 and TLR9 (**Supplementary Figure 9**), both of which utilize Btk for signaling. To test Btk function in the observed autoimmunity, *Btk^-/-^* (45) and *BBim^fl/fl^* mice were intercrossed (DKO). Deletion of Btk did not alter overall proportion of splenic B cells in the DKO mice (**Figure 9A**), however, B cell subpopulation distribution was altered and reduced some characteristic features of autoimmune pathology in the *BBim^fl/fl^* mice (**Figure 9**). Specifically the proportion of mature splenic FoB1 cells (IgM^lo^IgD^hi^) was decreased, but not of mature FoB2 cells (IgM^hi^IgD^hi^, **Figures 9B, C**), the proportion of immature transitional (T1, IgM^hi^IgD^lo/-^) B cells was increased (**Figure 9D**), whereas MZ (**Figure 9E**) and anergic B cells (An1 or T3, **Figure 9F**) was decreased in the DKO mice compared to *BBim^fl/fl^* or WT mice. These outcomes are consistent with our and others previous findings that loss of Btk selectively reduces FoB1 cells and affects An1 B cell survival (64). These cellular alterations was accompanied with reduced lymphocytic tissue infiltration in the DKO mice (**Supplementary Figure 2B**, right panels). There was a modest increase in the proportions of IL-6^+^ and IFNα^+^ B cells in the *BBim^fl/fl^* relative to control B cells (**Supplementary Figures 9A, C**). However, Btk deletion had no significant effect on the circulating cytokines tested including (**Supplementary Figure 10**).

We also noticed that Btk deletion decreased overall basal serum IgM and IgG2a (**Figure 9G**). To determine if overall reduction in the immunoglobulin levels in the DKO mice had an effect on the accumulation of autoAbs, we analyzed serum using autoantigen arrays. Results show that both IgM and IgG autoAbs were decreased in the DKO mice (**Figures 9H, I**). However, IgM autoAbs were reduced in both young and old (**Figure 9H**), IgG autoAbs were decreased only in the old *DKO* mice (**Figure 9H**). Taken together, these results indicate that Btk-dependent signaling likely contributes to aberrant B cell activation and autoimmune pathology in the *BBim^fl/fl^* mice. This interpretation is consistent with previous reports that overexpression of Btk transgene led to SLE-like autoimmune disease and expression of SYK, another B cell kinase is increased in B cells from lupus patients (63, 65, 66).

## Discussion

Self-reactive B cells arise routinely during BCR diversification and are purged to avoid autoimmunity by receptor editing, anergy and apoptosis. Failure of any of these mechanisms could cause autoimmune disease, however, the precise contribution of each of these tolerance mechanisms in eliminating autoreactive B cells and preventing autoimmunity is unclear. To examine the contribution of B cell apoptosis in preventing autoAb production and autoimmune disease, we rendered B cells tolerance-compromised by B cell-specific deletion of proapoptotic Bcl-2 family member Bim, a known regulator of immune tolerance in B and T cells (34, 37). Our longitudinal analysis of *BBim^fl/fl^* mice indicates that apoptosis contributes to the elimination of autoreactive B cells significantly enough that dysregulation of apoptosis leads to the development of autoimmunity. However, the manifestation of autoimmune disease becomes apparent only with age; although autoAbs and tissue damage can be detected at a relatively young age (< 6 months), the pathogenesis is clearly evident after six months. Nonetheless, our data shows that autoAbs against several prototypical autoantigens associated with SLE and SS are present in *BBim^fl/fl^* mice at elevated level compared to NODH2h4, a widely used autoimmune mouse model (**Figure 6**). One possible explanation of the delayed onset of autoimmunity in *BBim^fl/fl^* mice is that the events that trigger autoreactive B cell activation such as inflammation, innate and/or T cell mediated signals take a while to accumulate and manifest in older mice. Furthermore, *BBim^fl/fl^* B cells are not entirely resistant to apoptosis as they may employ other proapoptotic pathways including Noxa, Puma and Bmf as shown in BAK and BAX double knockout mice (43). These data are consistent with prior findings that autoreactive B cell hyperactivity and autoantibody production in autoimmune diseases involves B cell intrinsic innate TLR signals and adaptive T helper cell signals (*via* CD40) as well as homeostatic regulation by BAFF (23, 67–69) and that depletion of B cells is beneficial for patients with several autoimmune diseases including SLE, RA, SS and MS (70).

Bim is expressed in all tissues including hematopoietic cells and it is a key physiological facilitator of apoptosis in lymphocytes purging autoreactive B and T cells (37, 71). Prior gene targeting experiments have demonstrated that systemic deletion of the gene encoding Bim leads to a systemic SLE-like autoimmune condition in a mixed 129SV x C57BL/6 genetic background. Subsequent reports indicated that SLE-like autoimmunity in Bim-deficient mice gets much milder when bred to pure C57BL/6 background. One explanation for a milder autoimmune response and pathology in Bim-deficient mice is reduced functionality of immune cells. For example, T cells in Bim-deficient mice are defective in TCR-induced activation and IL-2 production due to impaired calcium signaling (72). Additionally, T cell development is impaired at the DN to DP stages in the thymus altering T cellular composition and repertoire in the Bim-deficient mice (73). Alternatively, global loss of Bim in the whole body reduces release of self-antigens from dead and dying host cells that trigger autoimmune response. The data presented here demonstrates that B cell-specific deletion of Bim in C57BL/6 background can lead to SLE and SS-like autoimmune disease with age. We hypothesize that Bim-sufficient T cells and other immunocytes are more potent in promoting B cell activation in *BBim^fl/fl^* mice contributing to progression of autoimmunity. Our data showing expanded B cell compartment is consistent with prior studies using mb-cre or CD23-cre mediated B cell specific Bim deletion (40). The B cell expansion does not appear to be entirely due to extended survival as *BBim^fl/fl^* B cells could proliferate *in vitro* and during an immune response *in vivo*. The delayed appearance and mild autoimmune disease in the *BBim^fl/fl^* mice may explain why autoimmunity escaped detection particularly in younger mice (39, 74).

The immune cell expansion in the *BBim^fl/fl^* mice was not limited to B cells; an increase in T cells also contributed to the splenomegaly and lymphadenopathy. In this regard, autoimmune disease observed in mice lacking Bim selectively in the myeloid cell-lineage was accompanied with B cell expansion (39). However, mice with a B lineage specific deletion of Bim, described here provide an opportunity to investigate B cell subset-specific role in the development of autoimmunity. While FoB cells were markedly increased, B1 and MZ B cells which are often associated with the development of autoimmunity (75), were not increased suggesting B cell subtype-specific function of Bim in autoimmune pathogenesis. It is possible that the B1 and MZ B cell subsets are changed phenotypically in *BBim^fl/fl^* mice and contribute to autoimmunity in the *BBim^fl/fl^* mice. Future experiments will determine the effector functions and localization of B1 and MZ present in *BBim^fl/fl^* mice. In addition to mature FoB cells, we observed an increase in immature T1 and anergic T3 (An1) B cells. Like immature B cells in the bone marrow, T1 B cells are targeted for negative selection in the periphery to remove autoreactive B cells (7, 11, 18). Furthermore, T3 anergic B cell population is a rich source of autoreactive B cells (76). An increase in these B cell populations may contribute to break in B cell tolerance in *BBim^fl/fl^* mice.

SS has been long thought to be a T cell mediated disease especially in the initiation of the autoimmune process within the submandibular SG, however, there is growing evidence that B cells play multiple pathophysiological roles and may be important in the development of SS (77, 78). We found that *BBim^fl/fl^* mice displayed particularly strong SS phenotype with lymphocytic infiltration of SG, consisting of B cells, plasma cells and Tfh cells (**Figures 2**, **4**). The *BBim^fl/fl^* mice displayed hypergammaglobulinemia and had elevated autoAbs, including anti-SSB and anti-SSA autoAbs that are characteristic of SS (**Figures 5**, **6**). These data suggest that break in B cell self-tolerance can initiate the autoimmune process that lead to SS-like autoimmunity. However, whether anti-SSB and anti-SSA specific B cell expansion and activation occurs with the help of T cells and/or depends on the second signal *via* the TLRs and BAFFR remains to be determined. Thus, our data reveal a novel role for B cells in the initiation and progression of SS. It is unclear how B cells initiate autoimmune reaction. One possibility is that this role is associated with *BBim^fl/fl^* B cell function as autoantigen presenting cells, as suggested by their ability to produce inflammatory cytokines like myeloid cells to initiate adaptive immune response (39). Further comparative studies may reveal distinct and overlapping functions of apoptosis resistant B and myeloid cells in autoimmunity.

We found that deletion of Btk reduced autoimmune pathology and autoantibody accumulation in *BBim^fl/fl^* mice (**Figure 9**). These data suggest that Btk contributes to the hyperresponsiveness of *BBim^fl/fl^* B cells to BCR and TLR signaling and differentiation into antibody producing cells. These data are consistent with appearance of SLE-like autoimmune disease in mice overexpressing Btk (65, 79), whereas pharmacological inhibition of Btk kinase by PCI-32765 decreased the disease symptoms in several autoimmune models (80). It is possible that loss of Btk function in myeloid cells contributed to the reduction in autoimmune pathology in the *Btk^-/-^BBim^fl/fl^* mice. Future myeloid-specific Btk deletion experiments will address this possibility.

We have demonstrated here that loss of Bim in B cells alone is sufficient to cause SLE/SS-like autoimmunity in C57BL/6 background, notwithstanding, delayed onset. We propose a model in which B cell-specific loss of Bim promotes autoimmunity in several ways. First, by allowing the survival of autoreactive T1 B cells that can go through maturation, damage host tissues by promoting activation of the innate and T cells leading to tissue immune cell infiltration, notably of the SGs and secrete autoAbs that possibly form immune complexes leading to kidney damage. Although, the B cells in *BBim^fl/fl^* mice primarily display prolonged survival, they may accumulate sufficient activation signals by self-nucleic acids over time and may present RNA/DNA complexed protein autoantigens to activate autoreactive T cells. This T and B cell interaction likely further promotes proliferation of immune cells and the release of inflammatory cytokines which reinforce the innate and humoral immune response to self-antigens as has been previously proposed (20). In support of this we observed an increase in mature FoB and tolerance susceptible T1 and anergic T3 B cells and the ability of *BBim^fl/fl^* B cells to undergo cell division *in vitro* in response to key immune response regulatory receptors, anti-IgM, BAFF-R and TLR, suggesting *in vivo* priming. Primed B cells in this model would be more responsive to unmethylated CpGs and RNA/protein complexes found in serum or apoptotic bodies from neighboring cells undergoing normal apoptotic processes in the Bim-sufficient mileue in the *BBim^fl/fl^* mice, fueling a feedback loop whereby B cell activation regulates the immune response to react against self-antigens. In support of this idea, genetic models have shown that impairment of effector cell apoptosis participates in the breakdown of tolerance through chronic signaling caused by repeated exposures to self-antigen resulting in autoimmunity (81). With the predisposition to autoimmunity, *BBim^fl/fl^* mice can serve as a model for interrogation of genetic and environmental factors that trigger B cell mediated autoimmune disease. Amelioration of autoimmune pathology by Btk deletion in our studies is consistent with our and others’ prior studies indicating contribution of altered B cell signaling to autoimmunity and support the therapeutic use of Btk inhibitors in autoimmune diseases (41, 42, 79).

## Data Availability Statement

The original contributions presented in the study are included in the article/**Supplementary Material**. Further inquiries can be directed to the corresponding author.

## Ethics Statement

The animal study was reviewed and approved by Office of the Vice Provost for Research Institutional Animal Care & Use Committee Office (IACUC) University of Miami FL 33136.

## Author Contributions 

JAW, CB, ESC, DL-R, GC, and WNK designed research; JAW, ESC, CB, DL-R, GC, LIC, and TM, performed research; JAW, ESC, CB, DL-R, GC ELG and WNK analyzed data; NHA and LIC performed pathology and JAW, CB, ESC, GC, ELG and WNK wrote the paper. All authors contributed to the article and approved the submitted version.

## Funding

This work was funded by National Institutes of Health grant R21AI088511-01 and Intramural Funding Program, Sylvester Comprehensive Cancer Center, University of Miami to (WK).

## Conflict of Interest

Authors GC, LC, and TM are employed by AstraZeneca.

The remaining authors declare that the research was conducted in the absence of any commercial or financial relationships that could be construed as a potential conflict of interest.

## Publisher’s Note

All claims expressed in this article are solely those of the authors and do not necessarily represent those of their affiliated organizations, or those of the publisher, the editors and the reviewers. Any product that may be evaluated in this article, or claim that may be made by its manufacturer, is not guaranteed or endorsed by the publisher.
