# Supplementary material for: Impaired B Cell Apoptosis Results in Autoimmunity That Is Alleviated by Ablation of Btk
Source: Front Immunol. 2021 Aug 26;12:705307. doi: 10.3389/fimmu.2021.705307 (PMC8427801; doi:10.3389/fimmu.2021.705307)
Supplement: Supplementary file 1 [file Presentation_1.pdf]

Impaired B cell apoptosis results in autoimmunity that is alleviated by ablation of Btk

Jacqueline A. Wright,[1 †] Cassandra Bazile[1†,] Emily S. Clark[1], Gianluca Carlesso[2], Justin Boucher[1], Eden Kleiman[1], Tamer Mahmoud[2], Lily I. Cheng[3], Darlah M López-Rodríguez[1], Anne B Satterthwaite[3], Eric L. Greidinger[6], Wasif N. Khan[1*]

Supplemental figure legends

sFigure 1: B cell-specific deletion of *Bcl2L11* gene

To generate B cell-specific *Bcl2L11* gene deletion, previously described C57BL/6 mice with the first 4 exons of *Bcl2L11* gene flanked by LoxP sites [38] were crossed with C57BL/6 CD19-Cre mice (Rickert 1997, The Jackson Laboratory Stock No: 006785), termed *BBim* $^{fl/fl}$ mice. CD19-Cre is expressed in the B-lineage from the early pro-B to mature B cell stages. (A) Confirmation of loss of Bim protein in $B220^{+}$ B cells by intracellular staining with anti-Bim antibodies and flow cytometric analysis. (B) Confirmation of loss of Bim protein in B cells by Western blot analysis. Bim protein levels in whole cell extracts from purified $B220^{+}$ B cells from WT and *BBim*$^{fl/fl}$ mice. The proteins were separated on an SDS-PAGE gel, transferred onto immunoblotting membranes and probed with anti-Bim antibodies by immunoblotting. The same membranes were probed with anti-p38 antibodies as a protein loading control.

sFigure 2. ***BBim*$^{fl/fl}$ mice display splenomegaly and lymphocyte infiltration into non-lymphoid organs**. (A) Images of spleens from 6 month old *BBim*$^{fl/fl}$ and WT mice. (B) Lymphoid infiltrates in lung and liver of *BBim*$^{fl/fl}$ mice, which is reduced upon Btk gene deletion. (C) Images of salivary glands (lower right quadrant) and H&E staining showing lymphocyte infiltration of the salivary gland in 8 month old *BBim*$^{fl/f}$ mice. (D) H&E (top panel) and Immunofluorescence (lower panel) staining of spleens from 13 month old *BBim*$^{fl/fl}$ and WT mice. (E) Survival curve shows earlier mortality of *BBim*$^{fl/fl}$ relative to WT mice.

sFigure 3 **Submandibular lymph nodes from *BBim*$^{fl/fl}$ mice have increased T follicular helper cells.** Submandibular lymph nodes from *BBim*$^{fl/fl}$ and WT mice were assessed by flowcytometric analysis for the presence of T follicular cells. (A-B) Contour plots showing gates for T follicular helper (Tfh) cells using (A) PD1 and CXCR5 and (B) PD1 and BCL6 expression on CD4 gated splenocytes. (C-D) Representative graphs showing cell numbers (left panel) and proportions (right panel) of (C) PD1+ CXCR5+ CD4+ T follicular cells and (D) PD1+ BCL6+ CD4+ Tfh cells from the submandibular lymph nodes of *BBim*$^{fl/fl}$ and WT mice. Tfh cells from Bim-/- mice are show for reference (Bottom panel in A and B). Data is representative of >3 independent experiments. *P ≤ 0.05, calculated by Students T- test.

sFigure 4 ***BBim*$^{fl/fl}$ mice have tertiary lymphoid structures (TLS) in proximity of their salivary glands and in abdomen.** *BBim*$^{fl/fl}$ mice were evaluated for the presence of TLS. (A) Image of TLS in the abdomen of a *BBim*$^{fl/fl}$ mouse (representative of more than 6 mice). H&E image analysis (right panel) shows small densely packed lymphocytes. (B) Representative contour plots showing immune cell composition of TLS structures from 2 mice. Representative of several mice, which showed varied immune cell composition.

sFigure 5. **Serum IgG1 and IgG3 antibody levels** in WT and *BBim*$^{fl/fl}$ mice. Quantification of serum IgG1 and IgG3 from mice of the indicated ages was determined by ELISA. (A) IgG and IgG3 in ≤ 12 weeks old mice (B) IgG and IgG3 in ≥ 12 weeks old mice. This data extends results in main Figure 5 on the same serum samples. Data is representative of >3 independent experiments. Not significant (N.S), P > 0.05, *P ≤ 0.05, calculated by Students T- test.

sFigure 6. **Higher frequency of anti-SSA IgG autoantibody secreting cells (ASCs) in the salivary gland-associated (SG) lymph nodes of old *BBim$^{fl/fl}$* mice.** Salivary gland associated lymph nodes from *BBim$^{fl/fl}$* and WT mice were assessed for the presence of (A) IgM and (B) IgG ACS against the listed autoantigens. Spleens of WT and *BBim$^{fl/fl}$* mice at different ages were assessed for the presence of (C) IgM and (D) IgG ASCs against the listed autoantigens. Not significant (N.S) P > 0.05, *P ≤ 0.05, calculated by Students T- test.

sFigure 7- ***BBim$^{fl/fl}$* splenic and peripheral blood B cells (PBL) are resistant to IL-21 induced apoptosis costimulated either via TLR7 or CD40.** Splenocytes from WT and *BBim$^{fl/fl}$* mice were incubated for 48 hours with TLR7 agonist (CL097) or anti-CD40 Abs in the absence or in the presence of IL-21 (25ng/ml) as indicated and, then assessed for (A) live cells (white) and dead cells (gray) distinguished using a fixable viability dye (Invitrogen). (B) Representative flow plots showing the gating strategy to analyze live (7AAD- Annexin V-), dead (7AAD+ Annexin V+) and apoptotic (7AAD- Annexin V+) PBL B cells treated as in (A). (C) Bar graphs displaying percentages of dead (White), live (Gray) and, apoptotic (Black) cells in WT and *BBim$^{fl/fl}$* PBL B cell cultures gated as described in (B). Not significant (N.S) P > 0.05, *P ≤ 0.05, calculated by Students T- test.

sFigure 8 **. mRNA expression profiles of select genes in B cells from *BBim$^{fl/fl}$* relative to *Bim$^{-/-}$* mice.** Total RNA from enriched splenic B cells was isolated from WT, *BBim$^{fl/fl}$*, *Bim$^{+/-}$* and *Bim$^{-/-}$* mice and subjected qRT-PCR analysis. Relative fold induction was normalized to 18S rRNA and calibrated to WT B cells. (A-B) Bmf and Btk mRNA expression is increased in ***BBim$^{fl/fl}$* relative to *Bim$^{-/-}$*** B cells. (B-C) Bcl-2 mRNA expression is decreased in the *Bim$^{-/-}$* B cells. (D-E) TLR7 and TLR9 mRNA remains unchanged. Data is representative of >3 independent experiments. Not significant (ns) P > 0.05, *P ≤ 0.05, **P ≤ 0.01, ***P≤0.001, calculated by Students T- test.

sFigure 9**. Increased TLR7 and TLR9 induced cytokines in *BBim$^{fl/fl}$* B cells.** B cells from WT and *BBim$^{fl/fl}$* mice were cultured with CPG or CL097 for 24 hours (A) IL-6 and (B) IL-10 production was assessed by flow cytometry intracellular staining of IL-6 and IL-10. (C-D) B cells (C) from WT and *BBim$^{fl/fl}$* mice were analyzed for intracellular TNFα and IFNα cytokines without any further stimulation (Top panel) or incubated PMA/Ionomycin for 4 hours to enhance cytokine detection (Bottom panel). MFI values are shown. from ex-vivo (upper panel) and PMA/Ionomycin stimulated (bottom panel) B cells. (D) DCs were treated as in A and B and TNFα. IFNα and IFNγ were detected by intracellular staining. MFI values are shown. Data is representative of >3 independent experiments. Not significant (N.S) P > 0.05, *P ≤ 0.05, calculated by Students T- test.

sFigure 10. **Circulating proinflammatory cytokines in *BBim$^{fl/fl}$* mice with or without Btk deletion.** (A-F) The indicated cytokines were measured in the serum of 10-12 week old WT, *BBim$^{fl/fl}$*, and *Btk$^{-/-}$BBim$^{fl/fl}$* double KO mice using a cytokine bead assay.

sFigure 1

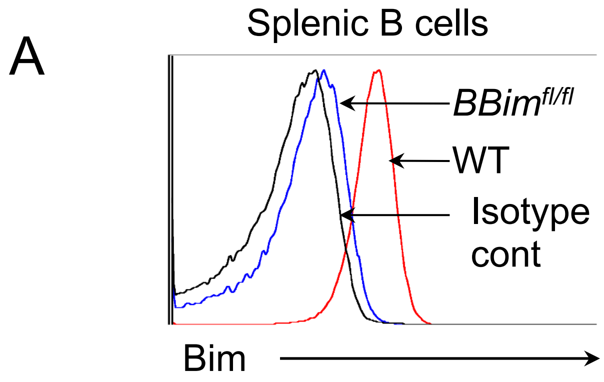
A
Splenic B cells
BBimfl/fl
WT
Isotype
cont
Bim

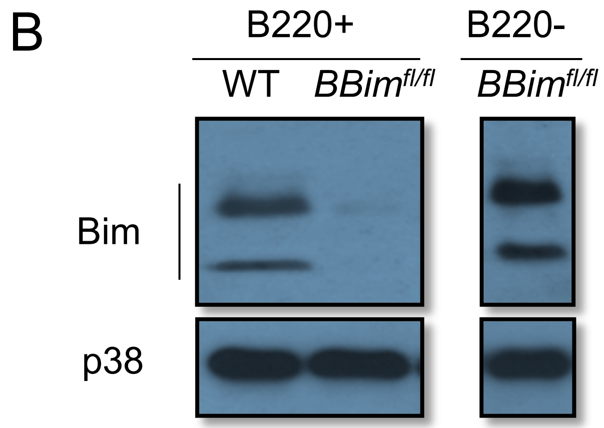
B
B220+
B220-
WT
BBimfl/fl
BBimfl/fl
Bim
p38

sFigure 2

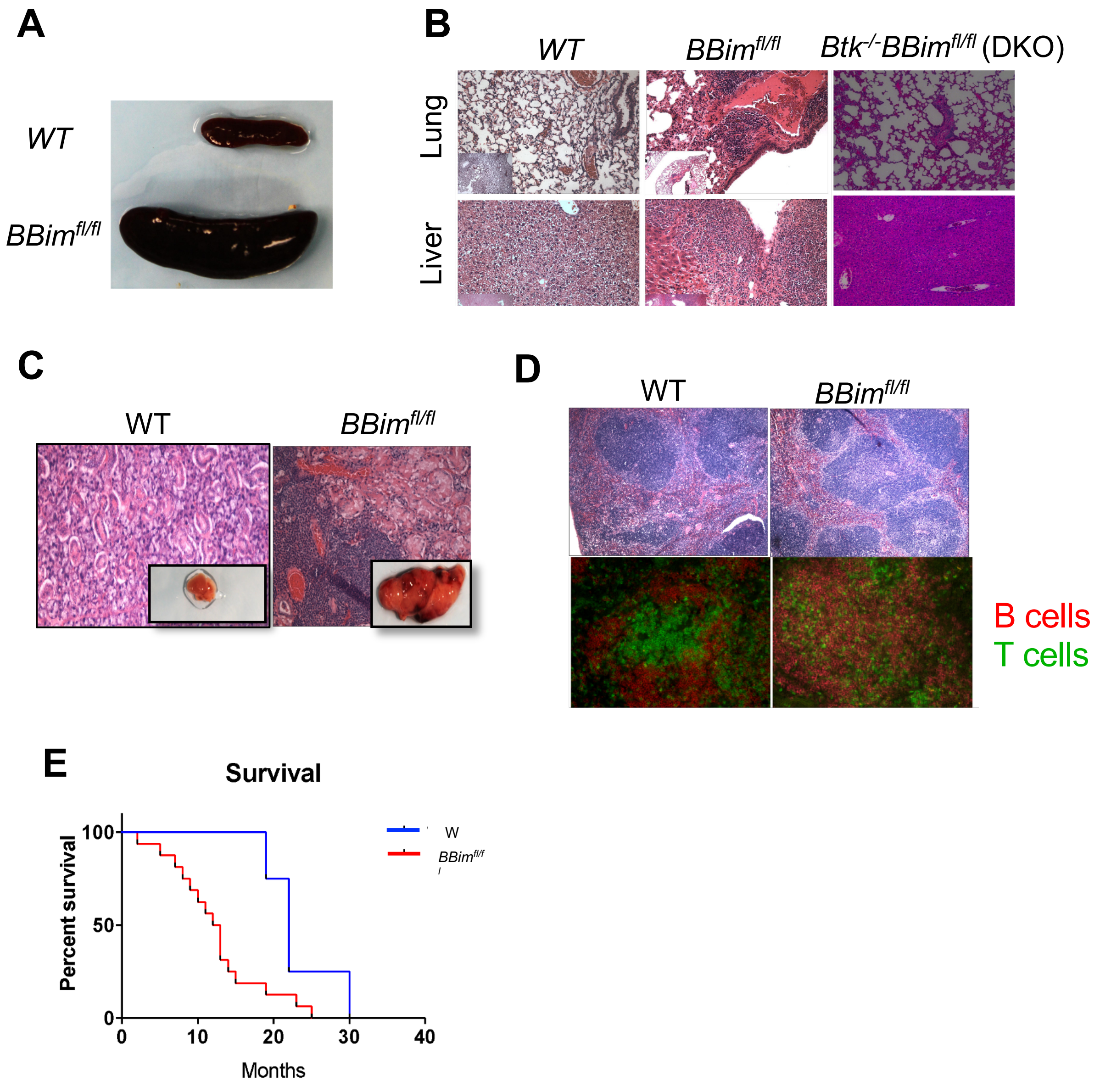
A
WT
BBim^fl/fl
B
WT
BBim^fl/fl
Btk^-/-BBim^fl/fl (DKO)
Lung
Liver
C
WT
BBim^fl/fl
D
WT
BBim^fl/fl
B cells
T cells
E
Survival
Percent survival
100
50
0
0
10
20
30
40
Months
W
BBim^fl/f
l

sFigure 3

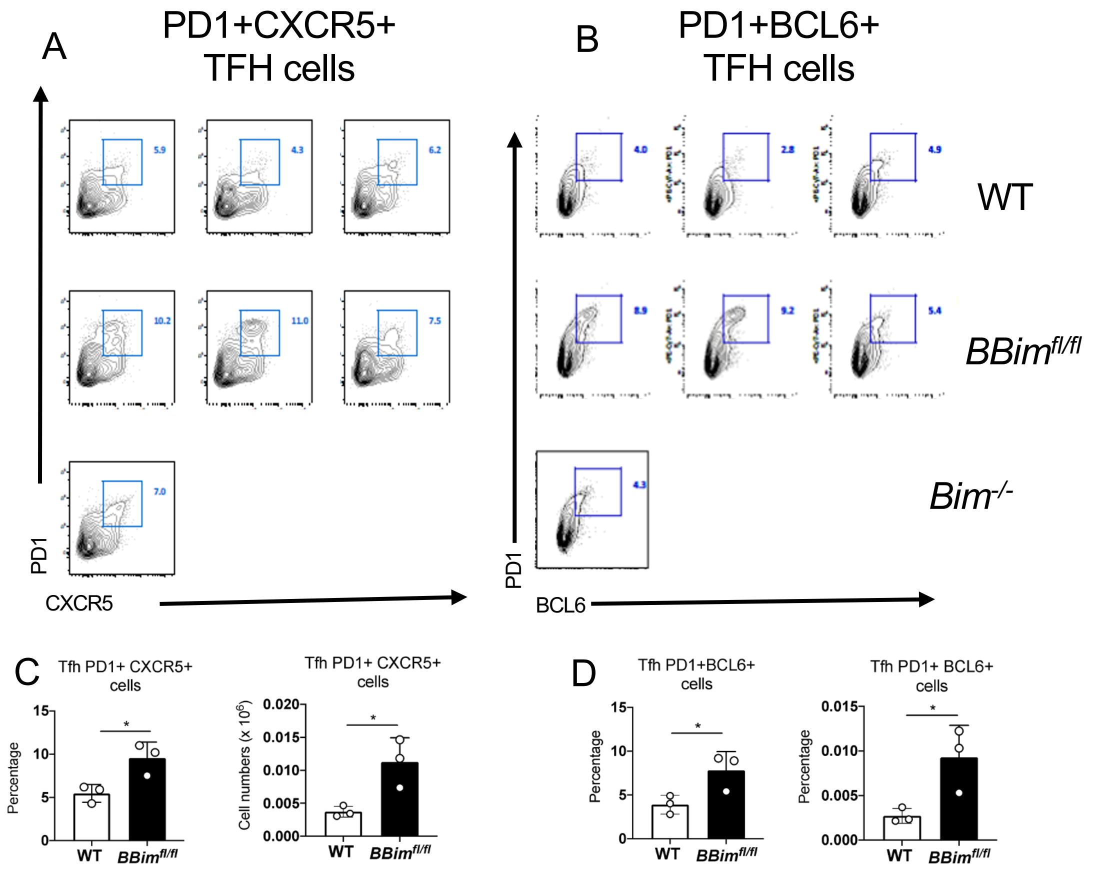
A
PD1+CXCR5+
TFH cells
5.9
4.3
6.2
10.2
11.0
7.5
7.0
PD1
CXCR5
B
PD1+BCL6+
TFH cells
4.0
2.8
4.9
8.9
9.2
5.4
4.3
PD1
BCL6
WT
BBim$^{fl/fl}$
Bim$^{-/-}$
C
Tfh PD1+ CXCR5+ cells
Percentage
WT
BBim$^{fl/fl}$
Tfh PD1+ CXCR5+ cells
Cell numbers (x 10^{6})
D
Tfh PD1+BCL6+ cells
Percentage
Tfh PD1+ BCL6+ cells
Percentage

sFigure 4

A

Tertiary lymphoid structures (TLS)

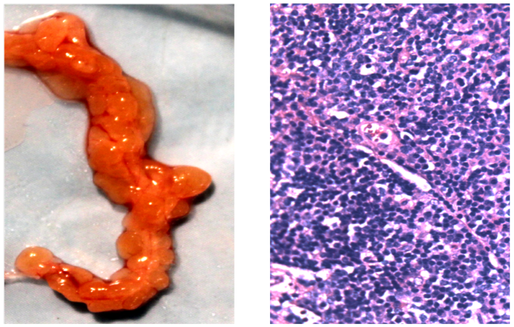

BBim$^{fl/fl}$

B

Immune cell composition of TLS

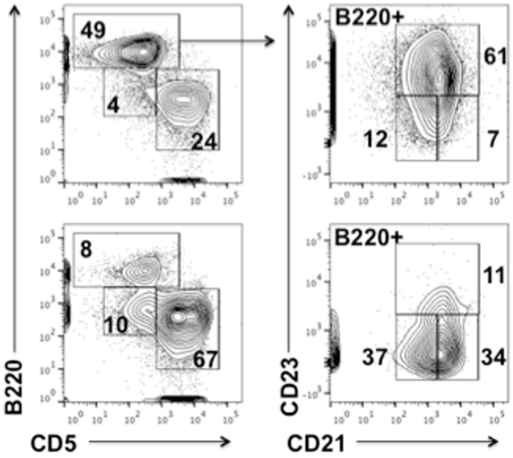

Mouse #1

Mouse #2

sFigure 5

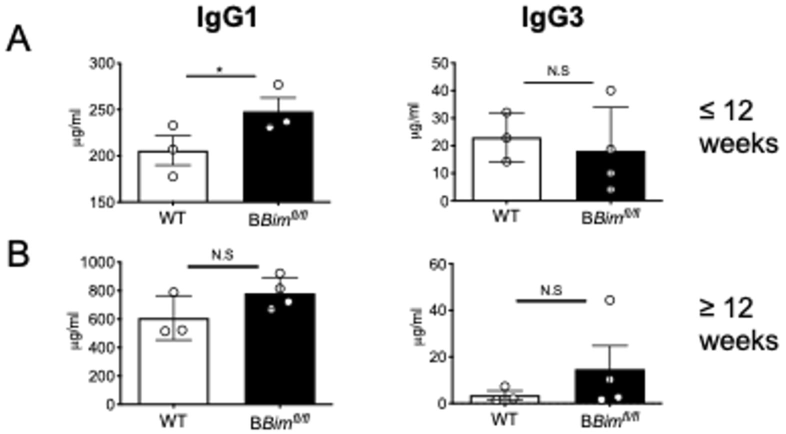
A
B
IgG1
IgG3
μg/ml
*
N.S
WT
BBim fl/fl
≤ 12 weeks
≥ 12 weeks
300
250
200
150
50
40
30
20
10
0
1000
800
600
400
200
60

sFigure 6

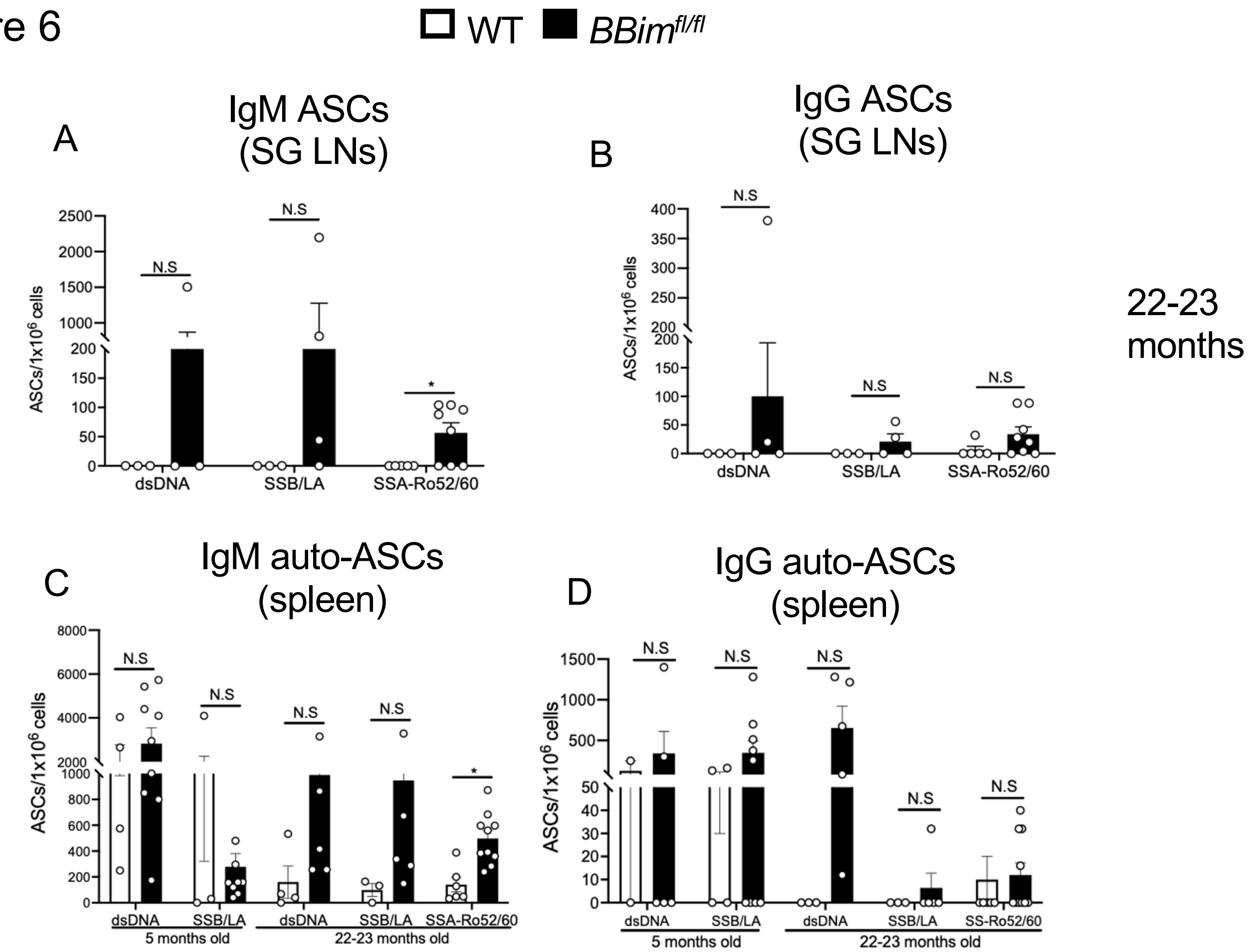
WT
BBim fl/fl
A
IgM ASCs (SG LNs)
B
IgG ASCs (SG LNs)
22-23 months
C
IgM auto-ASCs (spleen)
D
IgG auto-ASCs (spleen)
ASCs/1x10^6 cells
N.S
*
dsDNA
SSB/LA
SSA-Ro52/60
SS-Ro52/60
5 months old
22-23 months old

sFigure 7

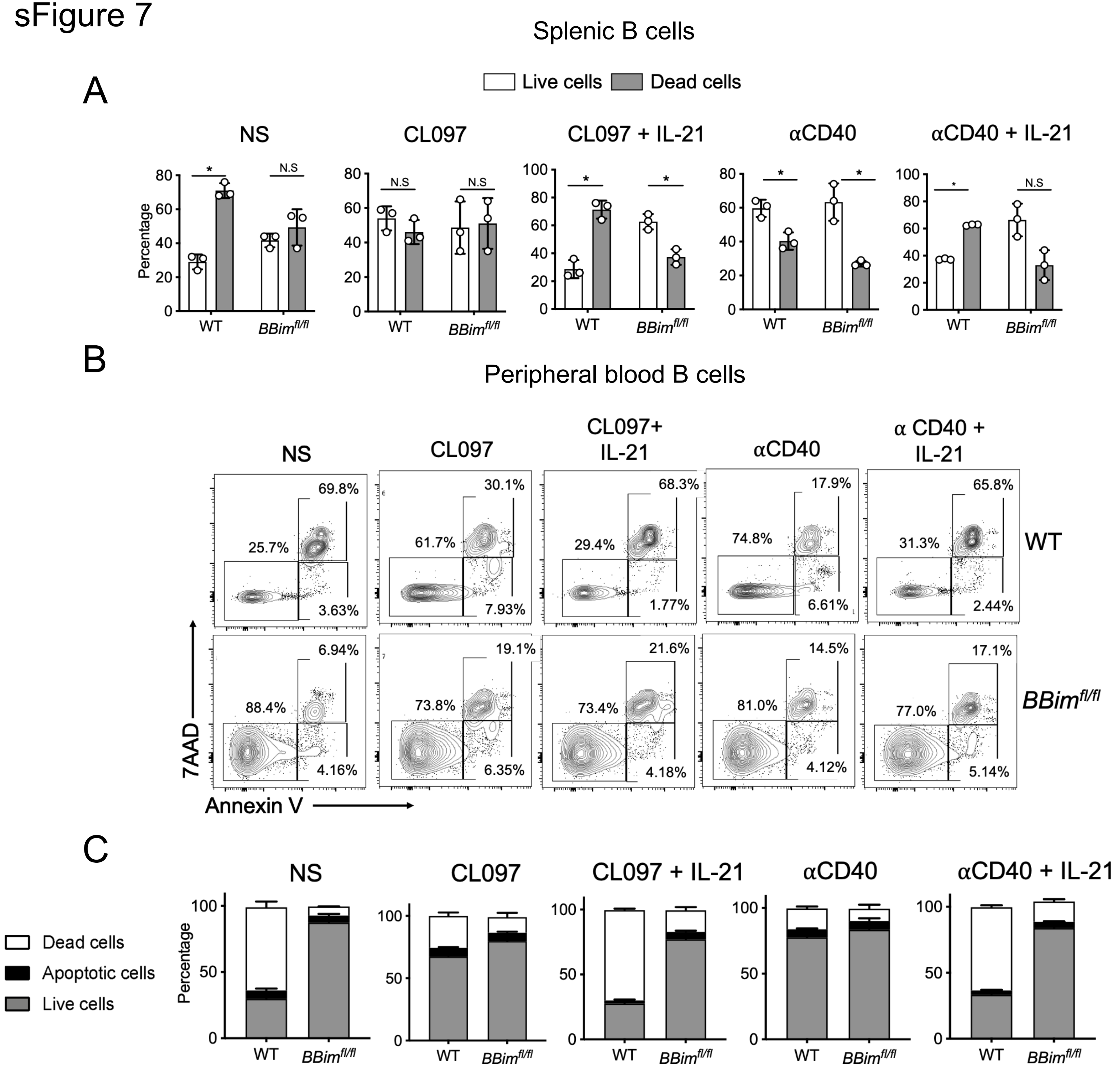

Splenic B cells
A
Live cells
Dead cells
NS
CL097
CL097 + IL-21
αCD40
αCD40 + IL-21
Percentage
N.S
WT
BBim^fl/fl
B
Peripheral blood B cells
NS
CL097
CL097+ IL-21
αCD40
α CD40 + IL-21
69.8%
25.7%
3.63%
30.1%
61.7%
7.93%
68.3%
29.4%
1.77%
17.9%
74.8%
6.61%
65.8%
31.3%
2.44%
WT
6.94%
88.4%
4.16%
19.1%
73.8%
6.35%
21.6%
73.4%
4.18%
14.5%
81.0%
4.12%
17.1%
77.0%
5.14%
BBim^fl/fl
7AAD
Annexin V
C
NS
CL097
CL097 + IL-21
αCD40
αCD40 + IL-21
Dead cells
Apoptotic cells
Live cells
Percentage
WT
BBim^fl/fl

sFigure 8

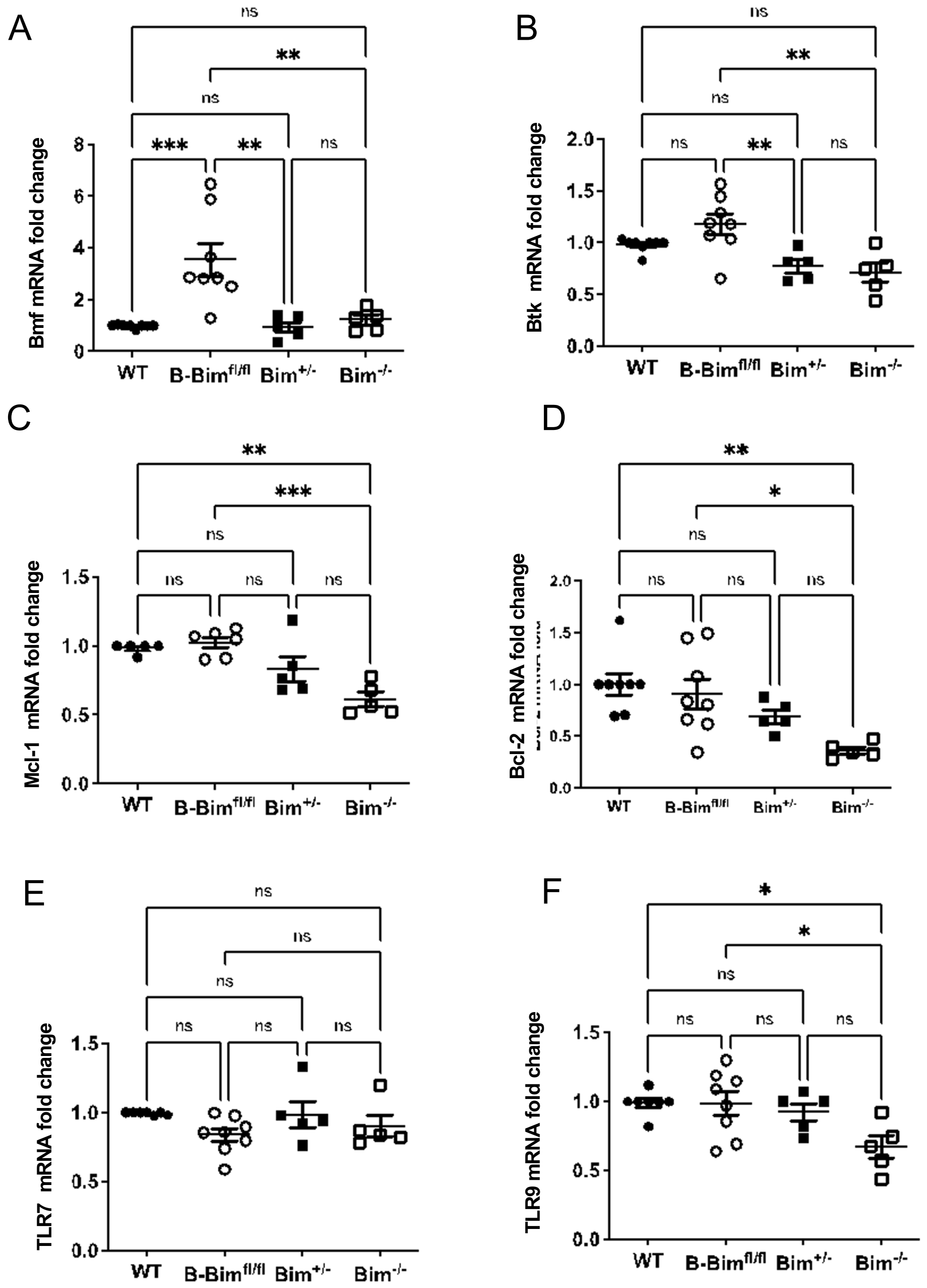
A
Bmf mRNA fold change
WT
B-Bim$^{fl/fl}$
Bim$^{+/-}$
Bim$^{-/-}$
B
Btk mRNA fold change
C
Mcl-1 mRNA fold change
D
Bcl-2 mRNA fold change
E
TLR7 mRNA fold change
F
TLR9 mRNA fold change
ns
*
**
***

sFigure 9

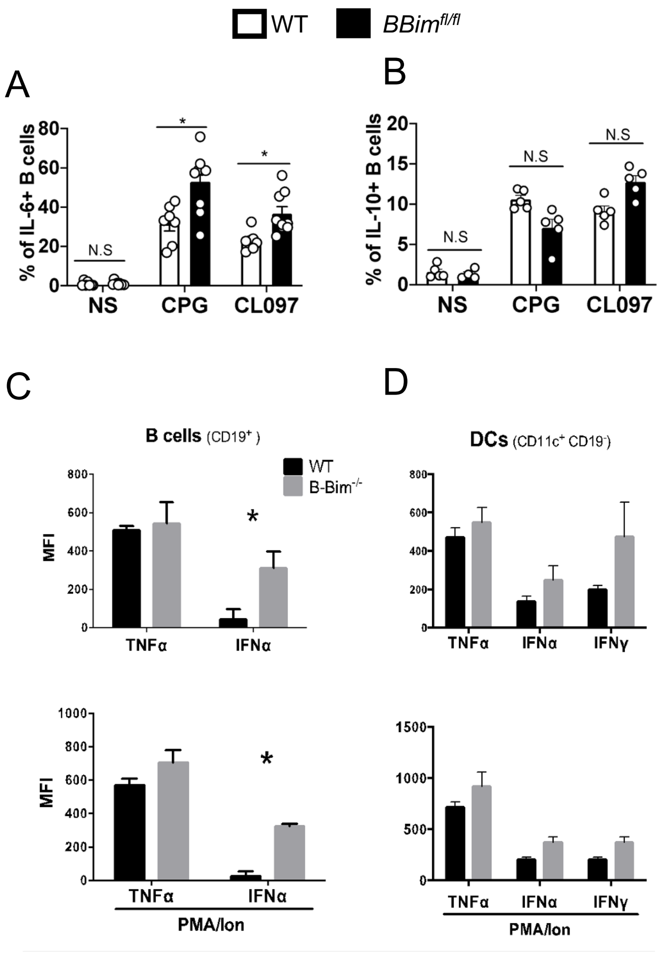
WT
BBimfl/fl
A
% of IL-6+ B cells
N.S
*
NS
CPG
CL097
B
% of IL-10+ B cells
N.S
NS
CPG
CL097
C
B cells (CD19+)
WT
B-Bim-/-
MFI
TNFα
IFNα
PMA/Ion
D
DCs (CD11c+ CD19-)
TNFα
IFNα
IFNγ
PMA/Ion

sFigure 10

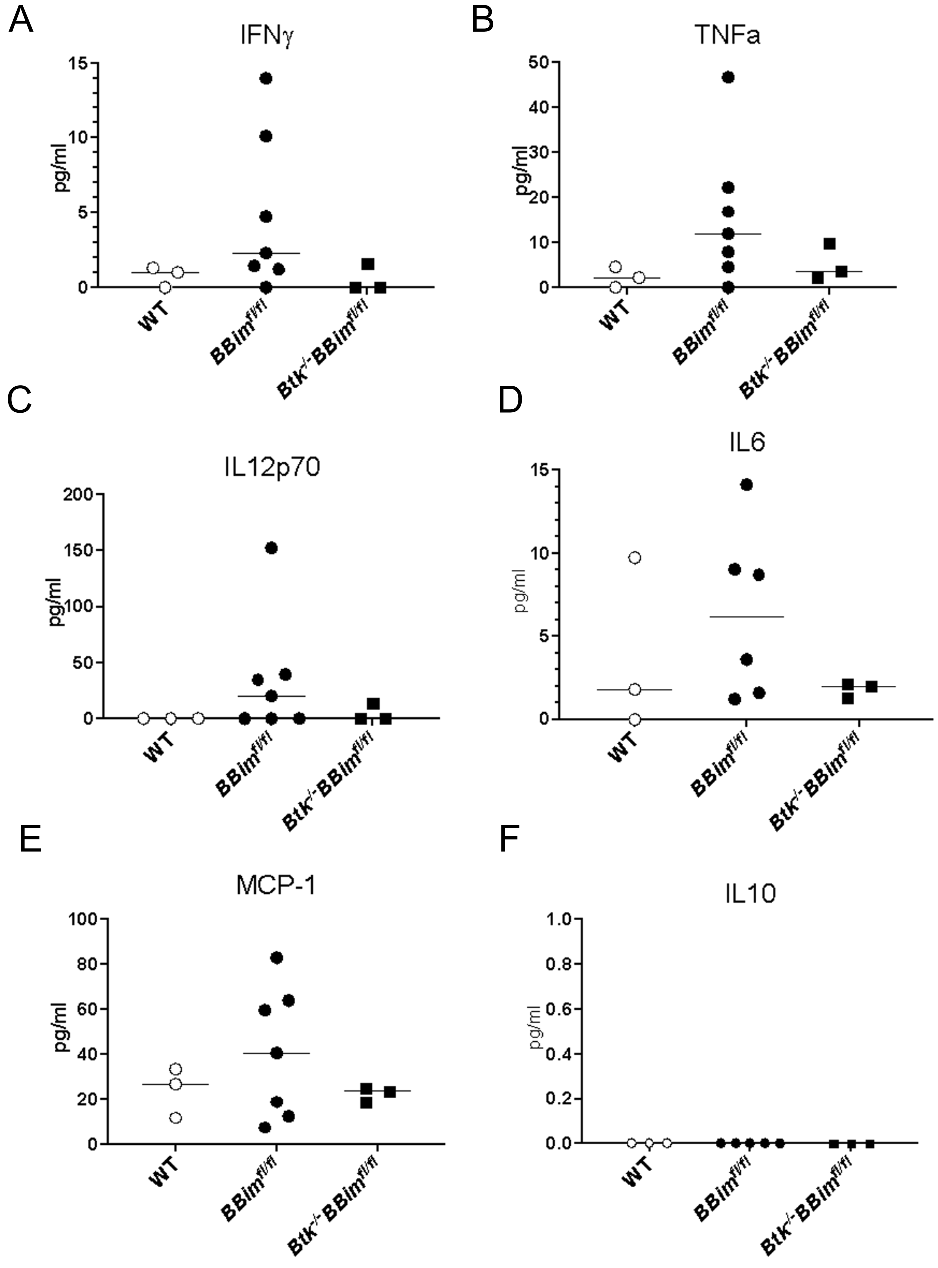

A
IFNγ
B
TNFa
C
IL12p70
D
IL6
E
MCP-1
F
IL10
pg/ml
WT
BBlm$^{fl/fl}$
Btk$^{-/-}$BBlm$^{fl/fl}$
